# Developing an integrated performance management and measurement system in healthcare organisations: a Canadian case study

**DOI:** 10.12688/f1000research.138430.1

**Published:** 2023-10-30

**Authors:** Anes Ben Fradj, Neila El Asli, Tasseda Boukherroub, Claude Olivier

**Affiliations:** 1 Systems Engineering Department, École de technologie superieure, Montreal, Québec, Canada; 2 Numérix Laboratory, École de technologie superieure, Montreal, Québec, Canada; 3 Interuniversity Research Centre on Enterprise Networks, Logistics and Transportation, Montreal, Québec, Canada

**Keywords:** AHP, BSC, performance management, healthcare

## Abstract

This study proposes a performance management and measurement system for a large healthcare organisation. First, data is collected to analyse and understand the current performance management system. Second, the SWOT (Strengths, Weaknesses, Opportunities, Threats) method is used to identify the main aspects of the performance management system to be improved. Third, based on the scientific literature and SWOT analysis, BSC principles are integrated to this performance management system to better align the organisation’s performance objectives and indicators with its strategy. Finally, we develop a performance indicator structure and specify indicators to be used as well as how these indicators could be integrated and shared with higher hierarchical levels in the organisation by using AHP (Analytic Hierarchy Process). Our approach is applied to the program “Physical disability, intellectual disability, and autism spectrum disorder” of CIUSSS du Centre-Sud-de-l’île-de-Montreal, a large healthcare network, in the province of Québec, Canada.

## Introduction

In the province of Québec, Canada, public health and social services are provided by networks of institutions called CI (U)SSSs (integrated (University) centres of healthcare and social services), which fall under the responsibility of the Ministry of Healthcare and Social Services (referred to as MSSS). Their main mission is to provide healthcare and social services to the population while ensuring accessibility, quality, safety, and efficiency. This study focusses on the CIUSSS serving the South-Centre of Montreal region (referred to as CCSMTL). It provides 28 different healthcare services to a population of 311,000 people, it owns 150 facilities and employs more than 21,300 people, including 56 senior managers and nearly 800 physicians. It manages a budget of more than $1.7 billion Canadian dollars ( Annual Report 2019-2020, CIUSSS). When the CCSMTL was created in 2015, it had to develop a reference framework for its performance management and to define a vision regarding its organisational performance. A *Management and Accountability Agreement*, involving the MSSS and the CCSMTL management, was introduced by the MSSS, with the obligation to implement a visual management system as a means for performance management and decision-making. Therefore, the CCSMTL has built its Quality-Performance Model (referred to as QPM), which encompasses four dimensions of performance; customer, accessibility/quality, mobilisation, and optimisation. It has also implemented its visual management system. However, QPM deployment is very challenging:

1) It must be implemented at different decision-making levels, organisational structures (programs, sub-programs, services, teams, etc.), and territories. For instance, performance indicators may differ greatly from one service to another or from one territory to another.
2) Some performance aspects such as customer and mobilisation dimensions are not sufficiently or well measured, which leads to an unbalanced performance management system.
3) CCSMTL managers need to measure the overall performance of a given service or program, through integrated information. Currently, such information is not available nor easy to obtain.

This study contributes to addressing the aforementioned issues. More precisely, it proposes a four-phases based approach to support CCSMTL managers to efficiently implement its QPM and provide them with an efficient performance management and measurement system (PMMS). In phase I, data is collected to analyse the QPM and the current performance management system. In phase II, the SWOT (Strengths, Weaknesses, Opportunities, Threats) method is used to identify the main aspects of this performance management system to be improved. In phase III, based on the scientific literature and SWOT analyses, BSC (Balanced Scorecard) principles are integrated to the CCSMTL performance management system to better align its performance objectives and indicators with its strategy. Finally, in Phase IV, we develop a performance indicator structure and specify indicators to be used as well as how these indicators could be integrated and shared with higher hierarchical levels in the organisation by using AHP (Analytic Hierarch Process). A real case study (Physical disability, intellectual disability, and autism spectrum disorder, referred to as DI-TSA-DP) proposed by the CCSMTL is used to illustrate our approach.

Among recent studies in the literature that reported on performance management in complex healthcare organisations such as the CCSMTL, we can cite Moisan (2019) who focussed on the CISSS of Gaspésie region (province of Québec). This study highlighted the experience of this institution in deploying its performance management system and described how it contributed to improve the client trajectory performance in the context of social services dedicated to youth. However, it is descriptive and does not propose a PMMS for the organisation. Patient-reported outcome and experience measures (PROMs and PREMs) for system performance measurement is considered as a way to enhance and promote the shift of healthcare systems towards a more coordinated, integrated and people-centred care. This approach has been the focus of recent studies such as Bull and Callander (2022) and Nuti *et al*. (2017). Nuti *et al*. (2017) discussed the importance of using patient-reported measures as well as indicators based on administrative data to evaluate cross-sectional healthcare services in a multidimensional healthcare performance system. Bull and Callander (2022) discussed the different performance measurement programs employed across England, the US and Australia, and suggested key recommendations for advancing PROM and PREM programs internationally. Our work contributes to the theory and practice by proposing a performance management and measurement approach adapted to large healthcare organisations such as CCSMTL.

The remainder of this paper is organised as follows: the next section presents the QPM in more detail. After that, we present our literature review and then describe our approach. Following, we present how the approach is applied to the DI-TSA-DP case and discuss the preliminary results. Finally we conclude in the last section.

### The quality-performance model

**Figure 1.**
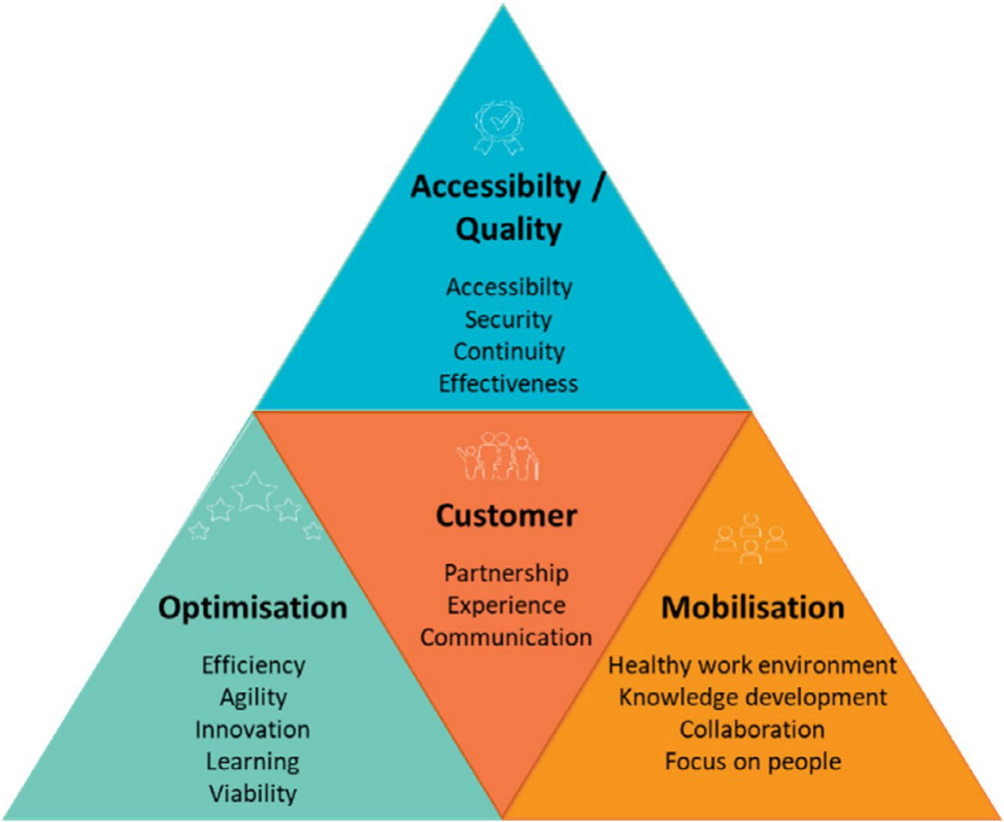
Quality-Performance Model (QPM) ( Couillard, 2018).

The QPM (see Figure 1) is based on the MSSS strategic plan (2029-2023 period) and framework for assessing the performance of the public healthcare and social services system ( MSSS, 2012), Accreditation Canada’s quality management framework ( Agrément-Canada, 2014), and PLANTREE’s person-centred approach ( Cosgrove, 1994). The QPM encompasses four dimensions of performance: customer, accessibility/quality, mobilisation, and optimisation. Each dimension presents an aspect of the CCSMTL performance and encompasses a number of sub-dimensions enriched by the perspectives of the customers and the community (see Table 1).

**Table 1.** QPM dimensions and sub-dimensions enriched by the customer and community perspective ( Couillard, 2018).

Dimension	Sub-Dimension	Definition
**Customer**	**Partnership**	Involve myself and my relatives as partners in the care and services you provide
	**Experience**	Make sure you have a human relationship with me so that I have a positive experience
	**Communication**	Listen to me, inform me of my rights and give me complete information in accessible language
**Accessibility / Quality**	**Accessibility**	Welcome me with an open mind and provide me in a timely fashion with appropriate care and services
	**Security (safety)**	Ensure my safety
	**Effectiveness**	Do what it takes to provide me with the best care and services
	**Continuity**	Coordinate my care and services throughout my trajectory
**Optimisation**	**Efficiency**	Avoid waste when using your resources
	**Viability**	Consider all perspectives to meet my present and future needs
	**Agility**	Be proactive and responsive to my expectations, values, and rights
	**Innovation**	Dare to introduce new ways to offer care and services
	**Learning**	Learn from experience how to do better
**Mobilisation**	**Healthy work environment**	Take care of those who care for me and humanise the physical environment
	**Focus on people**	Work with community partners who can help us
	**Knowledge development**	Support those who care for me to maintain and develop their knowledge
	**Collaboration**	Talk to each other and work together

Accessibility/quality and customer are the two main dimensions of the QPM. They are presented in the form of a compass needle that points in the right direction to achieve better performance. Positioned at the centre of the model, customer dimension focusses on customer satisfaction regarding services and care provided. It presents three sub-dimensions: partnership, experience, and communication. Customers refer to people who receive a service or care and their relatives, as well as the community and the population in general. Accessibility/quality is the organisation’s ability to meet safely the needs and expectations of customers and ensure accessible and continuous services. It presents three sub-dimensions; accessibility, safety, and relevance/effectiveness of services. Mobilisation is the use of skills and talents of all people working in the organisation as well as fostering personal achievement and commitment to fulfil collectively the organisation’s mission. It encompasses four sub-dimensions, efficiency, agility, innovation, learning, and sustainability. Finally, optimisation is the search for continuous improvement in order to use efficiently available resources and provide services that are adapted to customer needs. Its sub-dimensions include healthy work environment, knowledge development, collaboration, and focus on people.

The CCSMTL also implemented its visual management system formed by a network of control rooms at different decision levels of the organisation. Put simply, a control room is a dedicated space where managers and employees meet regularly to acquire information about current situation and initiate discussions to improve future performance ( Lagacé et Landry, 2016). At the highest organisational level, we find the strategic room, which is used by the CEO and senior managers. Data is used to evaluate the organisational performance, monitor current strategy, and support strategic decision-making (e.g. prioritising major projects). Tactical rooms are deployed in the different programs and sub-programs. Managers discuss their departments’ performance and decisions to be made at their level. Some of these decisions flow-up to the strategic room for validation while others flow-down to ‘intermediate’ tactical rooms or operational control rooms to be executed, in a cascade-escalation mechanism ( MSSS, 2015). Some tactical rooms referred to as “intermediate tactical rooms” might play an intermediate role between tactical and operational levels. Operational control rooms (or visual stations) ensure day-to-day operations management ( MSSS, 2015). The QPM is aimed at being implemented in all CCSMTL control room networks to support performance management.

However, as mentioned earlier, QPM deployment is very challenging for the CCSMTL. First, it must be implemented at different decision-making levels, organisational structures, and territories. However, performance indicators may differ greatly from one service to another or from one territory to another. Second, some performance aspects such as customer and mobilisation dimensions are not sufficiently or well measured, which leads to an unbalanced performance management system. Finally, CCSMTL managers need to measure the overall performance of a given service or program, through integrated information. Currently, such information is not available nor easy to obtain.

### Literature review

A performance measurement system could be seen as the process of assessing the organisation’s progress in achieving its goals ( Kairu *et al*., 2013). It contains financial and non-financial measures ( Okwo & Marire, 2012). It should enable the correct deployment of the strategic and tactical objectives and provide a structured framework enabling the relevant feedback of information to the appropriate levels to facilitate decision-making and control ( Bititci *et al.*, 1997). Botton *et al.* (2012) classified performance measurement systems according to three categories of approaches: strategy-oriented (BSC ( Kaplan & Norton, 1996), performance prism ( Neely *et al*., 2002)), hierarchical (performance pyramid), and process-based (macro-process model ( Brown, 2008)). The QPM can be considered as strategy-oriented. Due to its multi-dimensional and strategy-focussed aspects, it is consistent with the BSC model. In their recent systematic review, Amer *et al*. (2021) provided managers and policymakers with evidence to support using BSC in the healthcare sector. They conclude that BSC implementation demonstrated positive outcomes for patient satisfaction and financial performance. In their systematic literature review, Betto *et al*. (2022) attempted to understand the evolution of BSC in healthcare and their results revealed that the majority of studies focussed mainly on the BSC design process, rather than BSC implementation, use, or review. To Bohm *et al*. (2021), the composition of BSC development teams in healthcare should consider including patients as well as their families where appropriate and select highly achievable and valid performance measures aligned with the organisational strategy.

Developed in 1992 by Kaplan and Norton, the BSC is the result of a re-examination of performance measurement systems that usually give too much importance to the financial aspect. It is designed to measure four perspectives: finance (measures financial performance and the efficient use of financial resources), customer (concerns the organisation’s impact on the market in terms of customer satisfaction), internal processes (focusses on the efficiency and quality of internal processes) and learning & growth (concerns the culture and perspectives of the organisation, human resources, and information system). The term “balanced” underlines the concept of balance between all four axes of the model ( Kaplan & Norton, 1992). In BSC model, we distinguish leading indicators, which measure processes, and lagging indicators that measure outcomes. Causality is one of the most important aspects in the implementation of BSC: there is an implicit causal relationship between BSC perspectives and lag-lead indicators ( Porporato, Tsasis, et Vinuesa, 2017). To this end, a visual map (or strategy map) is built to show the objectives and causal links needed to execute a strategy. Scholey (2005) provides a well-structured six-step based process for creating a strategy map. Kaplan and Norton stressed the need to measure causal relationships, by using a complementary analytical approach ( Barnabè *et al*., 2012) even though, it is not easy to analytically validate all causal relationships ( Lorino, 2001; Schrage & Kiron, 2018). This is also the case for healthcare ( Porporato *et al*., 2017).

**Figure 2.**
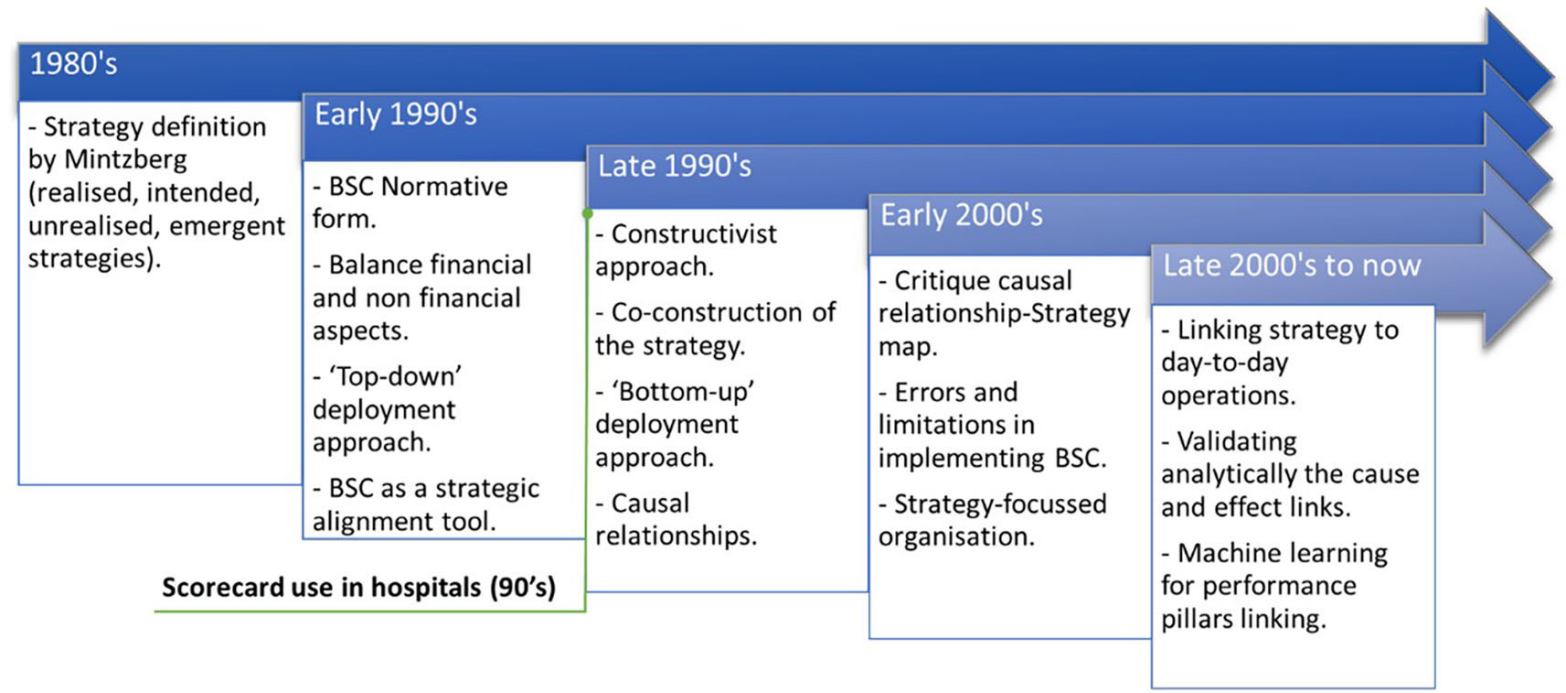
BSC evolution over time and application in healthcare.

Canada leads in hospital performance measurement and historically, it is among the first countries to develop scorecards in healthcare ( Porporato *et al*., 2017). In Toronto, Ontario, Women’s College Hospital started using a scorecard based on key objectives in the 1990’s ( Porporato *et al*., 2017). Since then, Ontario hospitals have gained great experience in designing and using scorecards. Chan (2009) presented the evolution of healthcare systems in implementing BSC and strategy maps in Ontario. From 1992 to date, BSC has evolved from a strategic alignment tool (normative form) to a strategy construction tool. The normative version proposes a “top-down” deployment approach to operationalise the organisational strategy. Studies have shown that BSC can contribute to the emergence of a new strategy, and it would be better to opt for a “bottom-up” approach, where strategic objectives would be the result of collaborative management ( Choffel & Meyssonnier, 2005; Ittner & Larcker, 2003; Norreklit, 2000; Simons, 1994). In addition, the learning & growth axis should aim to shape the organisation’s culture. Kaplan (2020) presented the case of merging St. Mary’s Hospital and Duluth Clinic (Minnesota, US) who co-created a new strategy, a strategy map and a common BSC. Montalan and Vincent (2011) proposed an approach for the co-construction of the strategy of a French hospital. They developed and validated a strategy map by a multidisciplinary teamwork following a bottom-up approach. Figure 2 shows the evolution of BSC over time and application in healthcare.

In order to measure the performance while considering the different dimensions of the BSC (healthcare and other sectors), some studies in the literature combined the BSC with multi-criteria decision-making techniques (MCDM). Most of these studies used the well-known Analytic Hierarchy (or Network) Process (AHP/ANP) ( Anjomshoae *et al*., 2019; Chan, 2006; Modak *et al.*, 2019; Regragui *et al.*, 2018; Yaghoobi & Haddadi, 2016). Chan (2006) combined AHP and BSC to identify global metrics and compare performance across Canadian healthcare organisations. Regragui *et al.* (2018) also combined BSC and AHP to develop a framework for assessing performance in hospitals. By using AHP, the authors weighted different performance perspectives and indicators, leading to a ranking of these indicators. In other studies, AHP has been successfully used to consolidate, prioritise, and weight performance indicators ( Bentes *et al*., 2012; Hossain *et al.*, 2019; Yaghoobi & Haddadi, 2016). For more on AHP applications in healthcare, see Hummel and IJzerman (2011). Recently, Daneshmand *et al*. (2022) developed a BSC-AHP based model for assessing the health system of the Social Security Organisation in Iran. They conclude that, the most important performance dimensions are internal processes and social obligations. Table 2 presents most recent BSC applications in healthcare including studies that combined BSC and AHP. Our proposed approach is inspired by the work of Regragui *et al.* (2018).

**Table 2.** Examples of most recent BSC applications in healthcare.

Country	Perspective	Reference
Australia	Validate a non-profit version of the BSC	( Soysa *et al.*, 2019)
	Integrating an environmental dimension with BSC	( Khalid *et al.*, 2019)
Brazil	Performance Management - survey on performance management practices in Brazil	( Portulhak *et al.*, 2017)
Canada	Performance measurement - implementation of a bottom-up approach	( Backman *et al.*, 2016)
	Integrating AHP to hospital scorecards in performance assessment	( Chan, 2006)
	Information management - key indicators - strategic alignment	( Nippak *et al.*, 2016)
	Causal Relationships	( Porporato *et al.*, 2017)
	Queue performance evaluation	( Breton *et al.*, 2017)
China	Establishing an Indicator System using the BSC	( Gao *et al.*, 2018)
Ethiopia	Performance measurement - development of a scoring methodology	( Teklehaimanot *et al.*, 2016)
Germany	Prioritise strategies	( Dekrita *et al.*, 2019)
India	Dynamic Balanced scorecard and its connectivity with Corporate Social Responsibility	( Ghosh & Singh, 2021)
Iran	Performance measurement system for the Social Security Organisation's health system.	( Daneshmand *et al.*, 2022)
Morocco	Conceptual framework for evaluating performance	( Regragui *et al.*, 2018)
Saudi Arabia	Cloud computing - Strategy map	( Alharbi *et al.*, 2016)
UK	Success factors for performance management	( Moullin, 2017)
USA	Accreditation in hospitals (ISO9001)	( Ritchie *et al.*, 2019)
	Merging health care organisations	( Kaplan, 2020)
Vietnam	Performance evaluation	( Pham *et al.*, 2020)

## Methods

**Figure 3.**
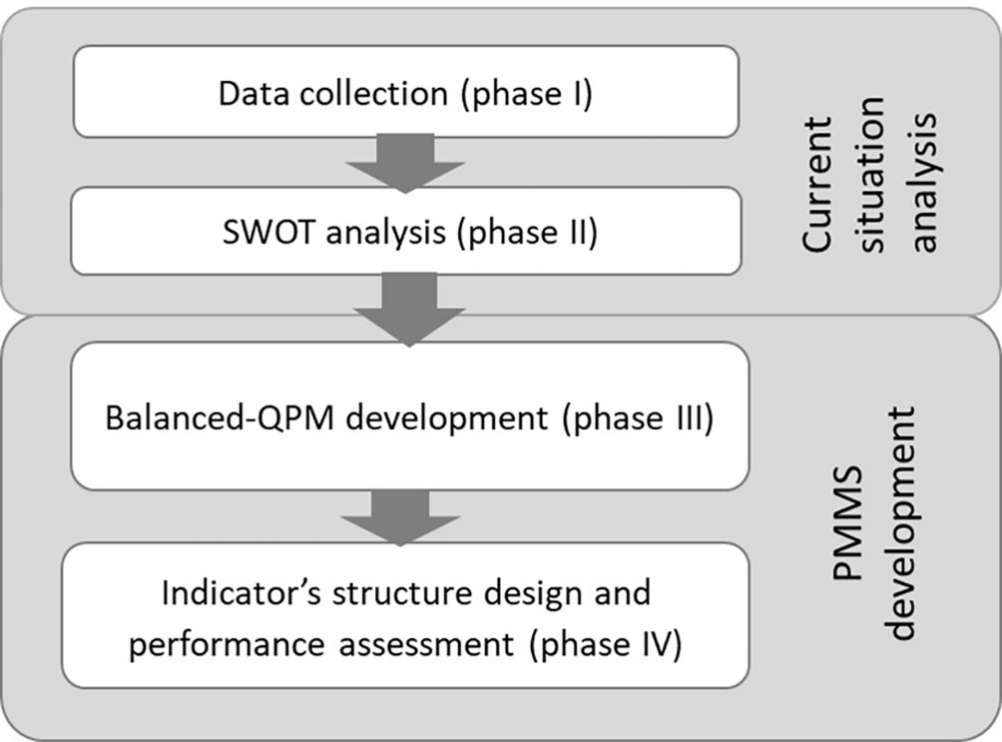
Proposed approach.

Our approach encompasses two parts as illustrated in Figure 3. The first part (Phases I and II) aims at describing and analysing the current situation while the second part aims at developing a PMMS (Phases II and IV).

### Data collection (Phase I)

The data collection phase began in May 2019 (the study started in January 2019). Collected data was used to gain a more in-depth understanding of CCSMTL internal processes and current practices and progress regarding the implementation and use of its visual management system and QPM. To this end, three methods were used: review of documents, field observations, and interviews.

Documents reviewed include MSSS’s Law on healthcare and social services, Health Standards Organization (HSO) standards ( HSO, 2021), Accreditation Canada ( Agrément-Canada, 2014), Ministerial reference framework for evaluating performance in public healthcare and social services systems ( MSSS, 2012), MSSS strategic plan 2019-2023, CCSMTL annual reports ( CCSMTL, 2016), and PLANETREE documents (e.g. Cosgrove, 1994). Regarding field observations, we participated in the QPM Coordinating Committee and the QPM Advisory Committee meetings. The Coordinating Committee, which is composed of a limited number of managers, aims at insuring the implementation of the QPM within the CCSMTL, in accordance with the MSSS strategic plan. Periodically, the Coordinating Committee invites the heads of all CCSMTL departments to discuss decisions to be made related to the QPM and collect their feedback. This enlarged group forms the QPM Advisory Committee. In addition, we participated in a performance improvement workshop and visited several control rooms of the CCSMTL at different levels of the organisation: the strategic room, five tactical and intermediate rooms, and 10 operational rooms. Finally, we visited two hospitals and two rehabilitation centres to learn more about day-to-day healthcare operations. A total of 12 interviews were conducted with 13 managers (from different departments/services and decision-making levels) between May 2019 and September 2021. The objective was to validate and consolidate our understanding of the performance management and measurement system and tools being used and the progress in implementing the QPM and visual management system. The structured interview was used ( DiCicco‐Bloom and Crabtree, 2006) (i.e., the interviewer asks several structured questions, and the interviewee responds freely to the questions). The participants were selected in collaboration with the deputy director of organisational performance at the CCSMTL, who is responsible for QPM implementation. If required, a second meeting was scheduled with the same participant(s).

### Data analysis (Phase II)

The SWOT method was used to structure and analyse the collected data. Our findings and conclusions are summarised in Table 3. These results have been validated by two senior managers of the CCSMTL involved in the QPM implementation.

**Table 3.** Results of the SWOT analysis.

	Strengths	Weaknesses
**Internal aspects**	- The QPM is well implemented at the strategic level. - Several practices ensuring high-quality services are implemented. - There is a willingness to embed a customer-focussed culture through the implementation of purposeful projects. - The visual management system is implemented at different decision-making levels. - There is a sufficient number of indicators within accessibility/quality dimension. - CCSMTL managers have a certain flexibility in proposing performance indicators for their departments. - There is good communication within the organisation.	- The QPM is known at the tactical and operational levels, but not used for performance management and measurement purposes. - The QPM dimensions are unbalanced in terms of number of indicators within each dimension. - There is a lack of global and aggregated indicators. - It is not possible for senior managers to easily examine the performance of a specific service or department. - There is a discontinuity in the assessment of performance between the hierarchical levels. - The information presented in dashboards is not well discussed. - Some indicators are interdependent, and other ones are conflictual.
**External aspects**	**Opportunities**	**Threats**
	- Several opportunities are provided by institutions such as PLANTREE, Agrément Canada, MSSS, etc. to implement best performance management practices.	- Socio-economic context (pandemics such as COVID-19 and population aging). - Workforce shortage issue.

One of the strengths identified is the well implementation of the QPM at the strategic level. There is strong interest by senior managers in aligning the organisation’s practices with QPM objectives. In addition, there are several practices put in place to ensure service quality and customer satisfaction and there is a real willingness to embed a customer-focussed culture through the implementation of purposeful projects. The deployment and use (even partially at the moment of conducting this study) of the visual management system at different decision-making levels demonstrates a real desire to build an effective management and measurement performance system. We also identified that there are enough indicators in the accessibility/quality dimension. Another important aspect is that managers at different hierarchical levels have the possibility to propose performance indicators in compliance with general ministerial objectives (even though some indicators are imposed by the MSSS). Moreover, each department holds regular meetings, which helps to maintain communication within the departments and support the information cascade-escalation process. While the QPM is well implemented at the strategic level, it is not sufficiently operationalised at the tactical and operational levels. We also identified that the QPM’s dimensions are unbalanced in terms of number of performance indicators used within each dimension. We find many indicators measuring the quality/accessibility dimension, but there is a lack of indicators in optimisation, mobilisation, and customer dimensions. There is also a lack of specific targets for those indicators. With the current performance management system, it is not possible for senior managers to track performance indicators at tactical/operational levels or to verify the overall performance without consulting a large amount of information. In addition, it is not possible to examine easily the performance of a specific service or department. There is a discontinuity in the assessment of performance between hierarchical levels. That is, each department/service measures its performance independently of the higher hierarchical level and other departments/services. We identified that dependencies and conflicts among some indicators exist, and this is challenging for managers to deal with. For example, there is a need to simultaneously reduce waiting time and improve direct service time (time with customers). An improvement in one indicator can decrease the other and vice versa. Regarding opportunities, the CCSMTL must continuously challenge itself to maintain/obtain accreditations and certifications such as PLANTREE, Accreditation Canada and to also comply with the MSSS’s strategic plans. Finally, like most organisations in a continuously changing socio-economic context, the CCSMTL might be threatened by pandemics such as COVID-19 or workforce shortage. These aspects can notably lead to disrupt service accessibility.

### Balanced-QPM development (Phase III)

Based on the results of our SWOT analysis and inspired by previous studies in the literature, we propose to use BSC principles to create a more balanced QPM. To build our balanced-QPM, the four axes of the BSC and the four dimensions of the QPM are compared to each other. General causal relationships in this new balanced-QPM are also explicitly represented. Finally, the generic strategy map of the CCSMTL is derived from the resulting balanced-QPM.

### Balanced-QPM dimensions and causal links

**Figure 4.**
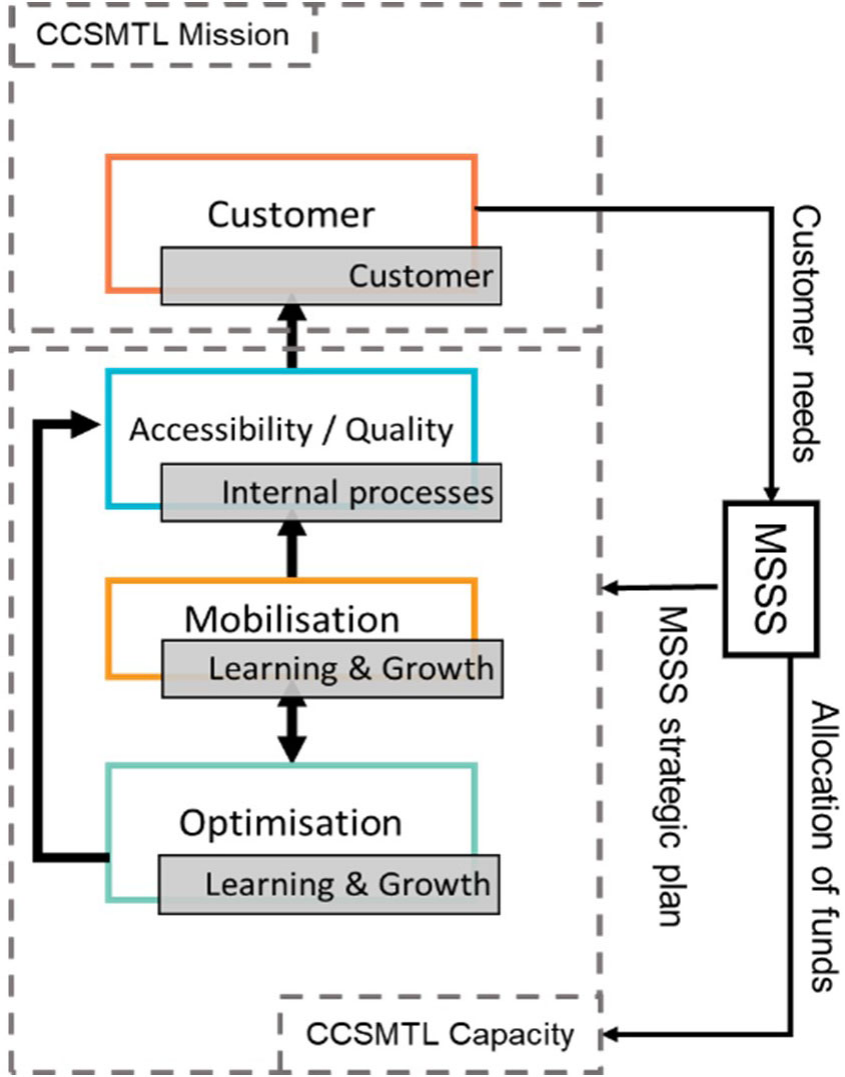
Proposed balanced-QPM.

Figure 4 shows the dimensions of the proposed balanced-QPM. BSC’s customer perspective corresponds to the customer dimension of the QPM. In the CCSMTL context, this dimension reflects the organisation’s mission and takes the first position in the balanced-QPM ( Figure 4). BSC’s internal process perspective is equivalent to the accessibility/quality dimension in the QPM. We recall that accessibility/quality refers to the ability to safely meet customer needs and expectations by providing accessible and continuous services. This dimension takes the second position in the balanced-QPM, forming with the customer perspective the “True North” of the CCSMTL. BSC’s learning & growth aspect is found in both mobilisation and optimisation dimensions of the QPM. Learning & growth presents three main components: human capital, information capital, and organisational capital. Human capital refers to the management of skills and behaviours of employees. This aspect is part of the mobilisation dimension of the QPM (use of skills and talents of everyone in the organisation, partners, customers, and their families, encouraging personal development and commitment to accomplish the mission of the CCSMTL). The mobilisation dimension takes the third position in the balanced-QPM. Organisational capital is defined as the organisation’s ability to align employee objectives with the strategy while information capital is the way organisations use their information systems, networks, and databases to support accomplishing their operations. Both aspects are part of the optimisation dimension of the QPM. We recall that optimisation dimension aims at continuous improvement and innovation to ensure, over time, that the services offered are adapted to the needs of customers. BSC’s financial perspective is also part of optimisation dimension. Kaplan and Norton ( Kaplan & Norton, 1999) stated that the financial perspective serves as a constraint, not an objective, for non-profit organisations such as healthcare systems. Therefore, optimisation dimension, takes the last position in the balanced-QPM.

Figure 4 shows that the customer dimension of the balanced-QPM corresponds to the organisation’s mission, and the other three dimensions to the CCSMTL’s capacity to fulfil this mission. Additionally, Figure 4 shows global causal links between the four dimensions. This aspect was absent in the current QPM. The optimisation dimension has direct impact on the accessibility/quality dimension. There is also a mutual relationship between optimisation and mobilisation. Mobilisation has an impact on the accessibility/quality of services provided by the CCSMTL, which in turn has direct impact on customer satisfaction. Finally, customers put pressure on the MSSS to provide the necessary funding supporting the CCSMTL capacity in terms of optimisation, mobilisation, accessibility/quality aspects.

### Strategy map development

**Figure 5.**
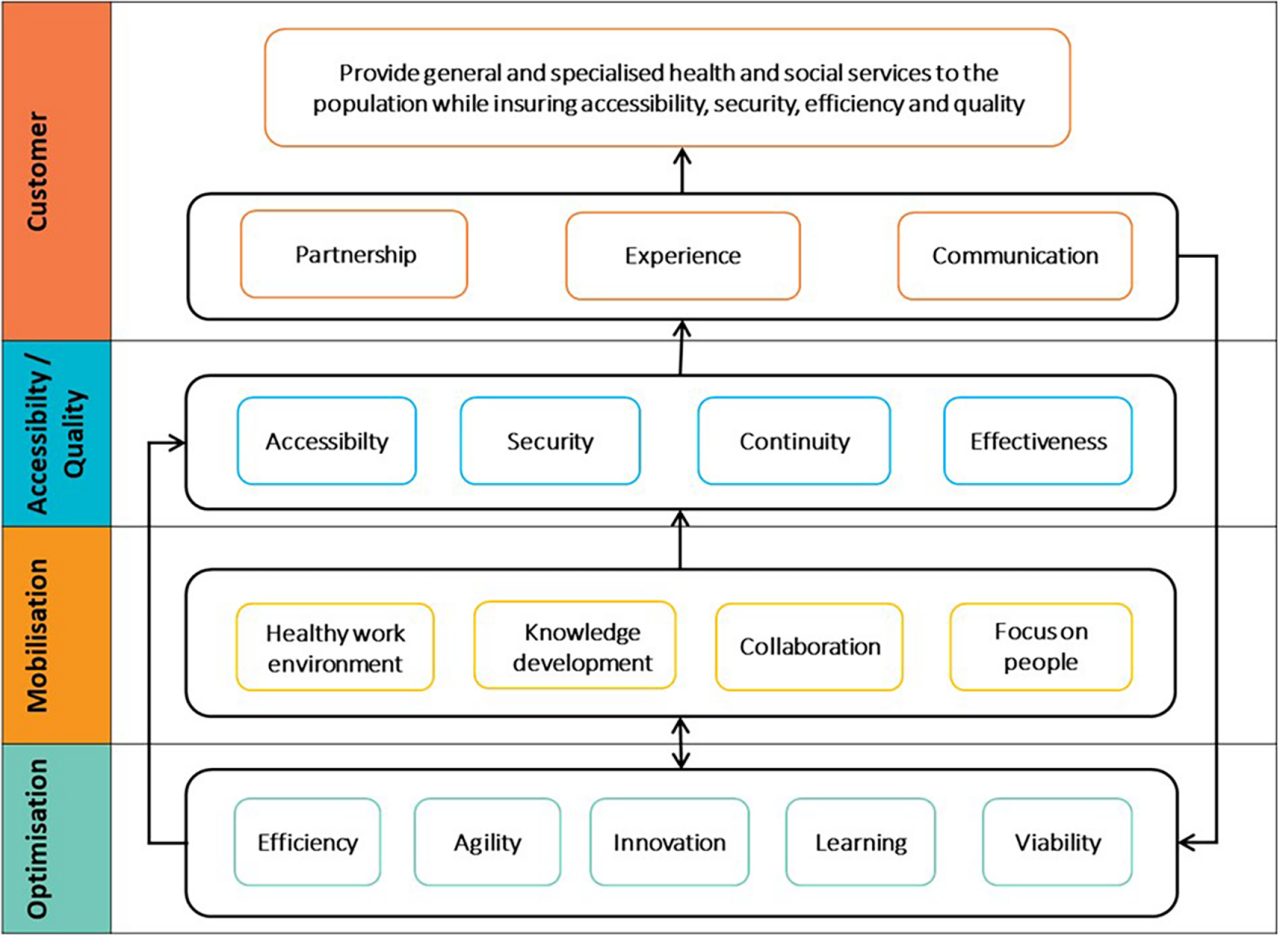
Generic strategy map proposed for the CCSMTL.

To build the CCSMTL’s strategy map, we focus on the six-step guidelines suggested by Scholey (2005) adapted to a non-profit organisation. The first step is to determine the primary objective, which is the desired end result once the strategy is implemented. For the CCSMTL, the primary objective is to satisfy its customers by providing general and specialised healthcare and social services while insuring accessibility, security, efficiency and quality. An example of a target to achieve for this primary objective within a specific time frame would be to increase the access delay compliance rate to 95% within one year. The second step is to select the appropriate value proposition. According to Treacy and Wiersema (2007), there are three value propositions for an organisation: operational excellence, product excellence and customer relationship. Since the CCSMTL follows a customer-focussed approach, its value proposal focusses on customer experience. The third, fourth, fifth, and last step aims at identifying the objectives of the customer, accessibility/quality, mobilisation, and optimisation dimension, respectively. The determination of these objectives can be guided by both the QPM sub-dimension definitions enriched by the customer perspective ( Table 1) and objectives set by the department managers, in relation to their specific mission. Rigorous criteria for selecting relevant objectives such as specificity, validity, sustainability, reliability, and utility were used to support this process. Figure 5 presents the generic strategy map of the CCSMTL that can be used as a basis for building “customised” strategy maps for different departments.

The causal links between the four dimensions shown in Figure 5 correspond to those identified in Figure 4. For the customised strategy maps, these causal links should be defined more precisely. This is illustrated with the DI-TSA-DP program case study. Validating analytically these causal links is beyond the scope of our study (time will be required to generate results and validate the causal links).

### Indicator structure design and performance measurement (Phase IV)

This phase aims at providing relevant indicators measuring performance following the objectives specified in the strategy map in each performance dimension.

### Step 1: Indicator system structure

**Figure 6.**
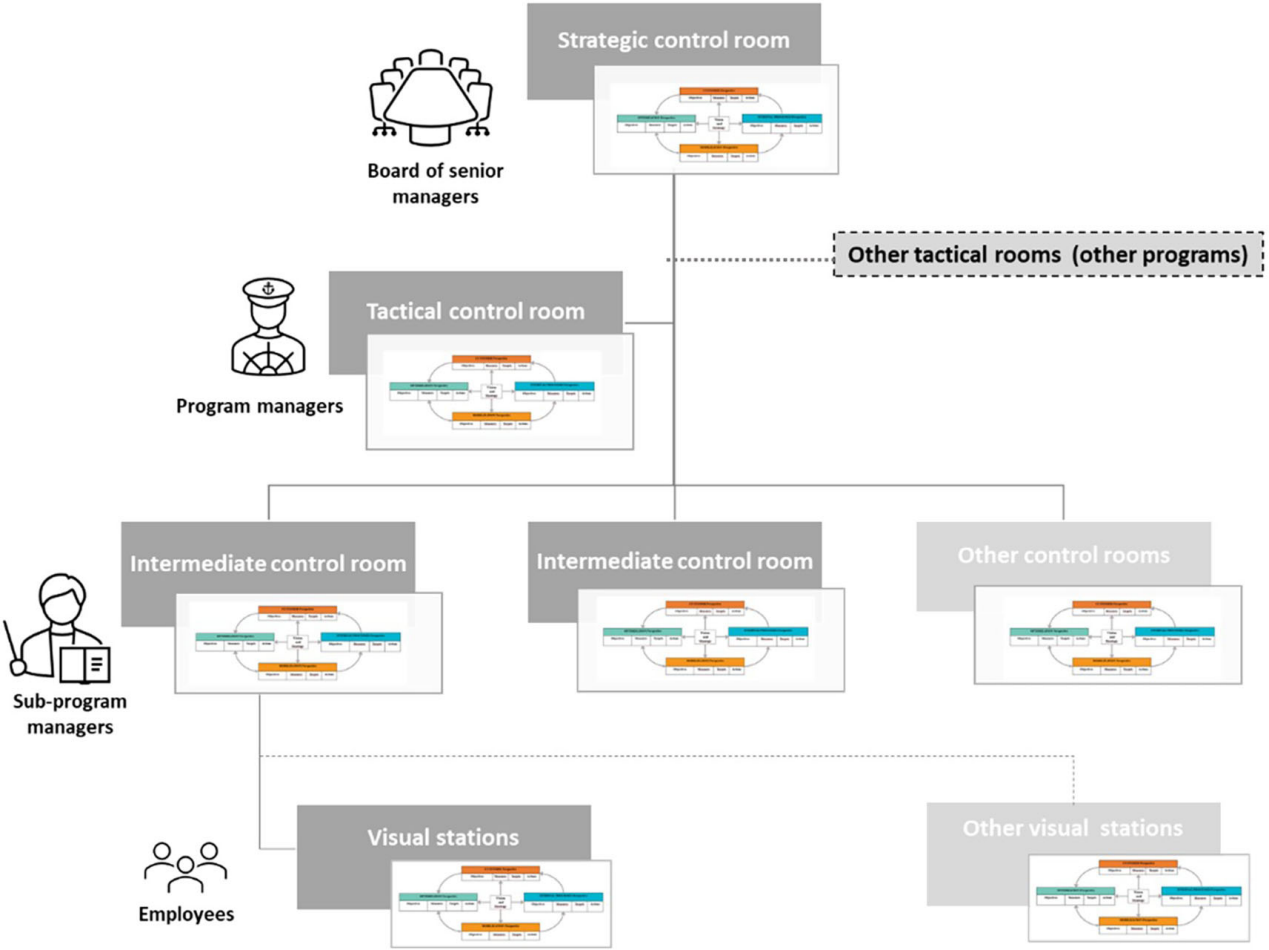
Proposed hierarchy and network-based scorecard structure. Balanced-QPM scoredcard at the visual station level.

We propose to create a structured system that provides indicators as well as performance indexes for each performance dimension at each hierarchical level of the CCSMTL. This general structure is based on the CCSMTL organisational chart and its visual management system. To provide and monitor the performance indicators, scorecards presenting the four dimensions (and their indicators) can be used at each department and hierarchical level, forming a network of scorecards (information is presented and discussed in the control rooms during regular meeting) ( Figure 6). This proposal is inspired from the study of Voyer ( Voyer & Voyer, 1999) that developed a network of interrelated dashboards used at different levels within an organisation. Béland and Abran (2004) also used this concept.

### Step 2: Indicator identification and selection

From the established strategy map, performance indicators are selected for each dimension following strategic objectives that are relevant to the department/service. Existing indicators should be reviewed first. Second, additional indicators are proposed according to criteria such as specificity, validity, sustainability, reliability, utility, etc. At the end of this step, each department will have a set of indicators for each dimension of the Balanced-QPM called “before normalisation indicators” ( $X_{\mathrm{bn}}$ ).

### Step 3: Indicator normalisation

The “before normalisation indicators” may have different units of measurement and different magnitudes. In some cases, the measurement unit is expressed in monetary units (e.g. expenses), and in others, in time units (e.g. hours) such as waiting time. In other cases, there is no measurement unit (e.g. number of services provided). In addition, the same indicator may have distinct targets in different departments. For example, waiting time target in an emergency department is significantly less compared to psychosocial services. Therefore, it might be difficult to compare them to each other based on their absolute values. Due to this, normalising the indicators is very helpful. Normalisation techniques for processing data have been used by many researchers in different fields. We refer the reader to Singh and Singh (2020) for more on normalisation. We selected the Min-Max normalisation technique ( Singh & Singh, 2020), mainly because minimum and maximum limits can be easily distinguished in available data provided by the CCSMTL. Following this technique, data is usually rescaled within the range 0 to 1 or (-1) to 1. The general equation is given as follows:

$$X_{\mathrm{norm}}=\left(\text{Nmin}-\text{Nmax}\right)\frac{X_{\mathrm{bn}}-\min}{\max-\min}+\text{Nmin}$$ (1)

$X_{\mathrm{norm}}$ : Normalised value of the indicator

$X_{\mathrm{bn}}$ : Indicator value before normalisation

min : Minimum value in the data

max : Maximum value

$\left[\text{Nmin},\text{Nmax}\right]$ : Normalised Min and Max

For the CCSMTL context $X_{\mathrm{norm}}$ is expressed as a percentage ranging from 0% to 100%, therefore Nmin et Nmax values are 0% and 100%, respectively. Given that in some cases the maximum value of an indicator is the undesirable value while the minimum value is the desired value (e.g. the maximum value for waiting time is non-desired while the minimum value is desired), we set the best value in the data set as max parameter (which is not necessarily the highest value) and the worst value in the data as min (which is not necessarily the lowest value) before normalisation. Outliers must be eliminated before choosing the best and worst values. To link a given indicator to a desired objective and to ensure its continuous improvement, max value corresponds to the indicator target. For each performance dimension, there is a set of associated indicators. Each indicator takes a numerical index i in the set and each indicator has an index *dim* referring to the first letter of the dimension to which it belongs (e.g. C for Customer). Indicators before normalisation take the index *bn*, referring to “before normalisation”. Normalised indicators in the original formula ( Equation (1)) $X_{\mathrm{norm}}$ become $X_{i}^{\dim}$ in the new formula adapted to the CCSMTL ( Equation (2)). We remove the index *norm* for the sake of simplicity.

$$X_{i}^{\dim}=\left(100\right)\times \frac{{\mathrm{Xbn}}_{i}^{\dim}-\min_{i}}{{\text{target}}_{i}-\min_{i}}$$ (2)

Each department/service at different hierarchical levels calculates its normalised indicators ( $X_{i}^{C}$ , $\;X_{i}^{A}$ , $\;X_{i}^{M}$ , $\;X_{i}^{O}$ ). These are the inputs of the fourth step.

### Step 4: Indicator weighting

**Figure 7.**
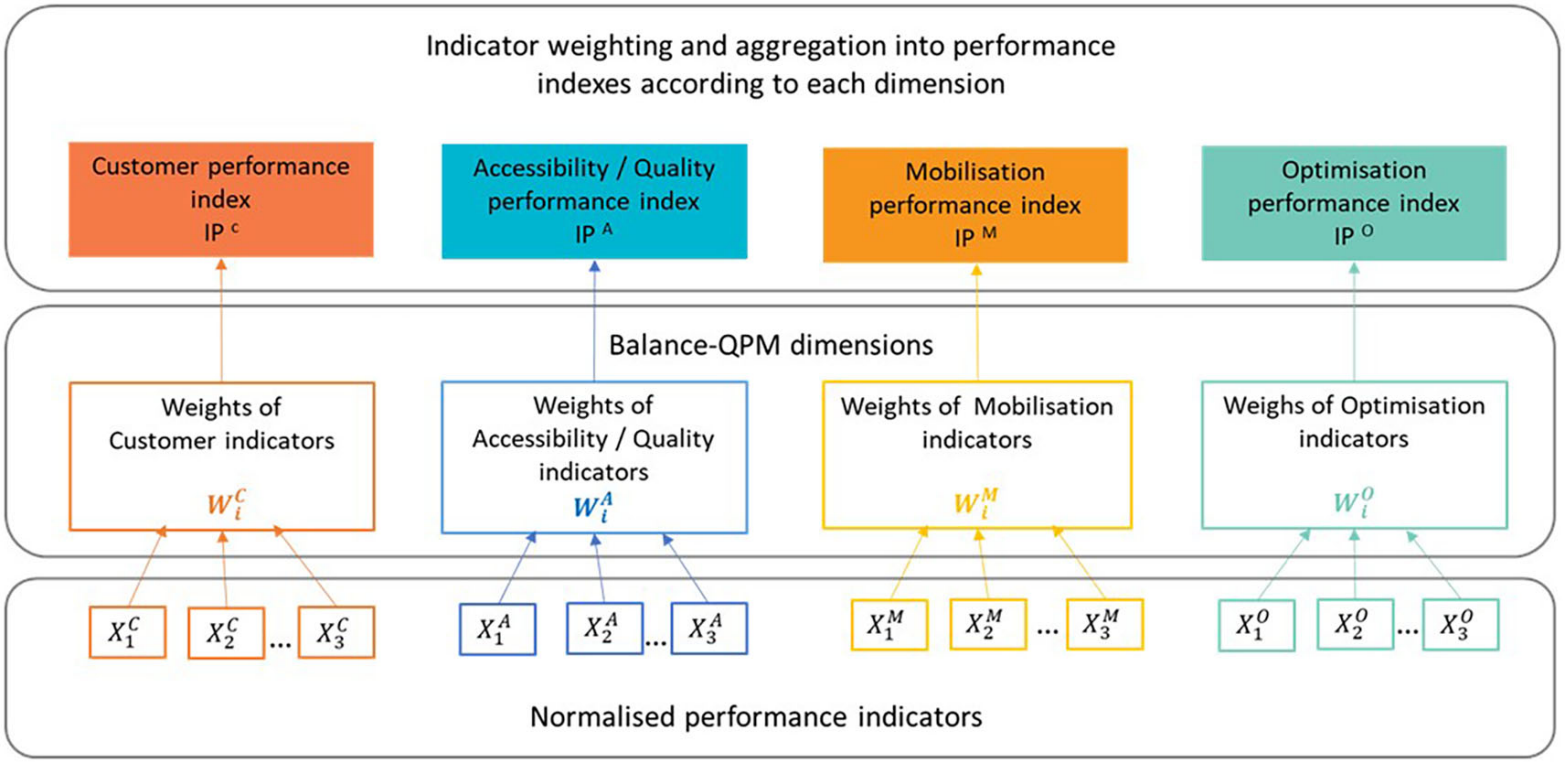
AHP adaptation for performance measurement for the CCSMTL case.

**Figure 8.**
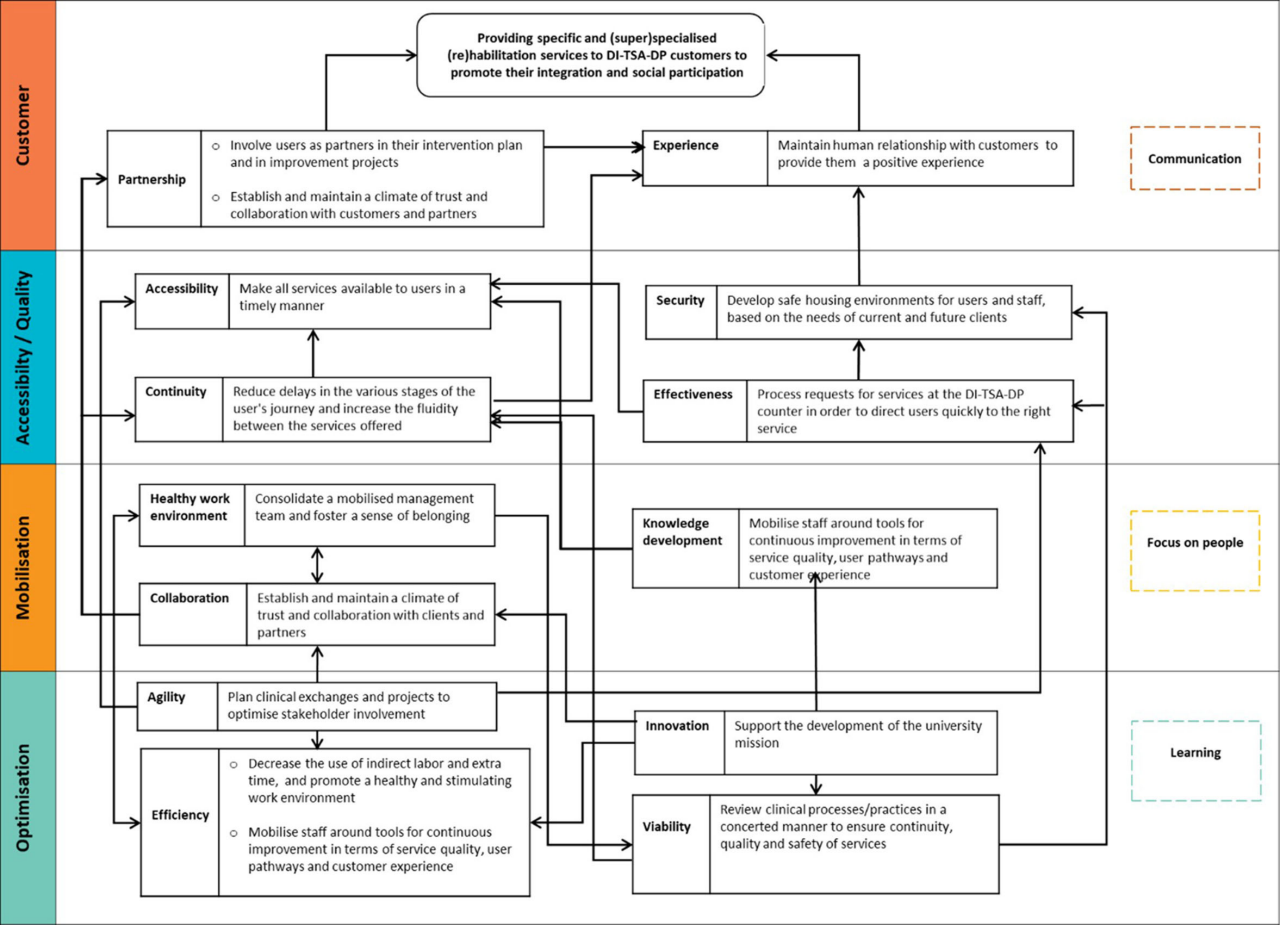
Proposed strategy map for DI-TSA-DP program.

Step 4 aims at weighting the normalised indicators of the same dimension at the same hierarchical level. AHP technique is used. AHP is usually used to evaluate and rank a set of evaluated based on several criteria. In the CCSMTL context, there is no need to rank or choose an option, but rather weighting the indicators within each performance dimension and aggregating them to obtain performance indexes for each dimension for a given department (see Figure 7). We selected AHP due its simplicity ( Kumar *et al*., 2017) since it is very convenient for decision-makers who are not familiar with analytical tools. Moreover, it relies on pairwise comparisons, and according to Ishizaka and Labib ( Ishizaka & Labib, 2011), psychologists argue that it is easier to express an opinion based on two elements rather than on all elements simultaneously. Figure 8 shows how AHP is adapted to our study.

Criteria and indicators that are usually used in AHP, correspond to the four dimensions of the QPM and to the normalised indicators $X_{i}^{\dim}$ , respectively. The normalised indicators are compared to each other by using pair-wise comparison matrices (one matrix for each dimension) according to their relative importance regarding the dimension to which they belong. These preferences are expressed by managers. A given element *a*_*ij*_ of a matrix A corresponds to the relative importance of indicator *i* (in the row) compared to indicator *j* (in the column). The preference values range from one to five, which is adapted from the preference scale proposed by Saaty (2005). For example, *a*_*ij*_ = 1 means that the indicators are equally important and *a*_*ij*_ = 5 that indicator *i* is extremely important compared to indicator *j*. The weights of the performance indicators are calculated by using a specific algorithm described in detail by Brunelli (2014). All AHP calculation steps are presented in Appendix A. Comparisons make sense only if the pair-wise comparison matrices are coherent or quasi-coherent. A consistency ratio (CR) is used to check this aspect. If CR is less than 10%, the matrix is consistent, otherwise, the pair-wise comparisons are repeated (for more details see equations 4, 5 and 6 provided in Figure 10). Once consistency is validated, the weights of the indicators, $W_{i}^{\dim}\;$ ( $W_{i}^{C},W_{i}^{A},W_{i}^{M},W_{i}^{O})$ are obtained.

### Step 5: Indicator aggregation

Indicators within each dimension are aggregated (see Figure 8), and four performance indexes $\left(P^{C},P^{A},P^{M},P^{O}\right)$ are calculated (one index for each dimension) following Equation (3):

$$P^{\dim}=\sum_{i}^{n}W_{i}^{\dim}\times X_{i}^{\dim}$$ (3)

$P^{\dim}$ : Performance index of dimension *dim*

$W_{i}^{\dim}$ : the weight of indicator *i* within the performance dimension *dim*

$X_{i}^{\dim}$ : i *th* normalised indicator within performance dimension *dim*

**DI-TSA-DP case study**

To illustrate how phases III and IV could be applied in practice, the “Physical disability, intellectual disability, and autism spectrum disorder” (DI-TSA-DP) program was proposed by the CCSMTL. First, DI-TSA-DP program is presented. Second, its strategy map is proposed (Phase III of our approach). Finally, the preliminary results of Phase IV (PMMS deployment) are presented, and a short discussion concludes this section.

### DI-TSA-DP program

The CCSMTL encompasses nine programs of which three; physical disability (DP), intellectual disability (DI), and autism spectrum troubles (TSA), are grouped together to form the joint program DI-TSA-DP. It provides services to 2,200 customers in local community service and rehabilitation centres. It receives approximately 10,000 service requests yearly and it encompasses 2,150 employees and 58 physicians. It includes three main sub-programs; DI-TSA, which provides services to customers with an intellectual disability or autism spectrum disorder, DP, which provides services to customers with a physical disability, and RMVS, which provides rehabilitation services in substitute living environments such as elderly homes. DI-TSA-DP mission is to provide specific, specialised and super-specialised habilitation and rehabilitation services to its customers to promote their integration and social participation.

**Figure 9.**
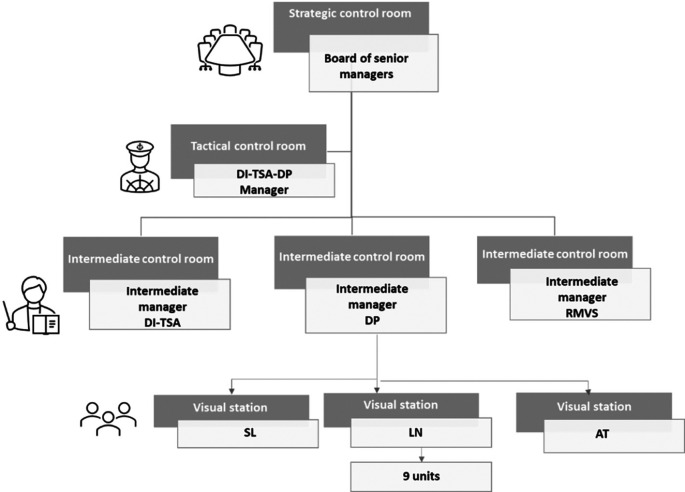
Structure of DI-TSA-DP program and its visual management system.

At the moment of conducting this study, DI-TSA-DP has put in place one tactical room (top management level of the program), and three intermediate control rooms at the sub-program level (DI-TSA, DP, and RMVS). Operational rooms are established at the team unit level within the three sub-programs. Control room deployment at the team unit level was still in progress. Therefore, each sub-program had only reached a certain deployment rate, which is seen as a measure of performance-culture maturity level in the CCSMTL. Following the recommendation of our collaborators, we focussed on DP sub-program, since it had the highest deployment rate. The approach can be applied to the two other sub-programs as well. DP includes three services: AT (Technical Aids), which provides special assistance devices (prosthesis, wheelchair, etc.), LN (Locomotor-Neurology), which provides services to customers with physical disabilities due to neurological or locomotor disorders, and SL (Sensory-Language), which provides services to customers with linguistic or sensory disabilities. We focus on LN service, again based on its operational room deployment rate (100%). There are nine care team units within LN service, and each unit has its own operational room. We worked in close collaboration with two teams, Locomotor team (A-BOG), which provides services for customers with an amputation or serious orthopaedic injuries, and Neurological team (AVC), which provides services for customers who have had a stroke ( Figure 9).

Prior to the application of our approach to DI-TSA-DP, we organised five meetings with DI-TSA-DP managers (strategic and tactical levels) in order to understand the specific mission and structure of DI-TSA-DP, collect data regarding the QPM deployment within the program, and describe our approach to the managers. These meetings also enriched the SWOT analysis (see Table 3). One conclusion was the absence of a well-structured performance indicator system within DI-TSA-DP. Moreover, there were no explicit links between the QPM dimensions, sub-dimensions, and DI-TSA-DP strategic objectives. Existing objectives were set independently from the sub-dimensions and performance indicators were not explicitly related to these objectives. The strategy map built for DI-TSA-DP presented in the next paragraphs contributes to addressing these lacks.

### DI-TSA-DP strategy map

The proposed strategy map for DI-TSA-DP ( Figure 8) is consistent with the generic strategy map ( Figure 5). It was co-created with the DI-TSA-DP program executive manager.

It illustrates objectives determined following the QPM sub-dimensions (including the primary objective) and causal links established between these objectives and/or sub-dimensions. The objectives selected are based on the internal DI-TSA-DP strategic vision 2018-2021 provided by DI-TSA-DP executive manager. Each objective was checked against the sub-dimensions of the QPM to identify to which one it is related. In total 16 objectives were classified within the sub-dimensions and dimensions of the QPM. The DI-TSA-SP mission was chosen as the primary objective and added in the customer dimension. Since no objective exists for customer experience, a new objective was proposed based on customers’ definition of this sub-dimension ( Table 1). Additional objectives should be set for this sub-dimension and other ones that currently do not present any specific objectives (i.e., communication, learning and focus on people).

Examples of causal links in the strategy map, are the relationship between objectives within customer sub-dimensions (‘partnership’ and experience’) and the primary objective (DI-TSA-DP mission). It is clear that positive customer experience, customer involvement as a partner in the system, and providing a climate of trust for the customers contribute to achieving DI-TSA-DP mission. It is interesting to notice that causal links also exist between sub-dimensions/objectives within the same dimension. As an example, partnership objectives contribute to achieving customer experience objectives. That is, when customers are involved as partners in the system, or when a climate of trust and collaborations is established with them, they are likely to have a positive experience. Some objectives/sub-dimensions mutually impact each-other, e.g. efficiency (optimisation dimension) and healthy work environment (mobilisation dimension). Reducing indirect labour and extra working hours (efficiency sub-dimension) leads to a healthy work environment, which in turn has a positive impact on efficiency.

### Indicator structure design and performance measurement

We conducted five working meetings of two hours each with different DI-TSA-DP managers. We started with the operational level (A-BOG and AVC) and moved to the tactical level (LN and DP service and sub-program, respectively), and finally, to the highest level (DI-TSA-DP program). The following paragraphs present the process of applying the developed PMMS to DI-TSA-DP case.

### Step 1: Indicator system structuring

Figure 9 shows a simplified organisational chart of DI-TSA-DP program. This flowchart shows only the departments needed to illustrate our approach. We distinguish four levels: 1) CCSMTL strategic level, 2) tactical level, which includes DI-TSA-DP program and DI-TSA, DP, and RMVS sub-programs, and 3) operational level (within DP sub-program) that includes SL, LN, and AT teams.

Figure 9 also shows the network of control rooms deployed in these different levels. The performance indicators structure follows the organisational chart and control room network structure. They are presented in the form of scorecards following the four QPM dimensions (indicators, values and targets, performance indexes).

### Step 2: Indicator identification and selection

To apply the rest of Phase IV steps, LN service was considered, and the process was performed in collaboration with LN coordinator. First, indicators being used by LN service were classified according to the four dimensions of the QPM and the strategy map’s sub-dimensions/objectives. Next, we checked the relevance of each performance indicator (against the criteria specificity, validity, simplicity, relevance, sustainability, reliability, and utility). When an indicator does not meet all criteria, it is either changed for another indicator that satisfies the criteria, or more information is gathered to ensure that the indicator meets all criteria. As an example, indicator ‘Customer satisfaction rate’ ( ${\mathrm{Xan}}_{1}^{C}$ ) ( Table 4), which was proposed to measure customer experience sub-dimension objective, did not meet simplicity criterion. LN managers did not have information on customers’ satisfaction and did not put in place specific methods to collect this information. To address this issue, we relied on the results of a survey used to collect the feedback of customers regarding their satisfaction level regarding experience and service quality. Table 4 shows the 10 final indicators selected for LN service.

**Table 4.** Indicators selected and their values after normalisation (LN service).

Dimensions	Measured sub-dimensions / Objectives	Indicator description	Indicator notation ( ${\mathrm{Xbn}}_{i}^{\dim}$ )	${\mathrm{Xbn}}_{i}^{\dim}$ value	Targets (Targeti)	Worst value (min _i_)	Normalized indicator ( $X_{i}^{\dim}$ ) value
**Customer**	Experience	Customer satisfaction rate	${\mathrm{Xbn}}_{1}^{C}$	75%	90%		75%
	Experience	Complaint rate	${\mathrm{Xbn}}_{2}^{C}$	60%	40%	100%	66.7%
**Accessibility/Quality**	Accessibility	Bed occupancy rate	${\mathrm{Xbn}}_{1}^{A}$	85%	95%		85%
	Accessibility Continuity Effectiveness	Respect of access deadlines	${\mathrm{Xbn}}_{2}^{A}$	80%	90%		80%
	Continuity	Average length of stay (DMS)	${\mathrm{Xbn}}_{3}^{A}$	17 days	10 days	50 days	82.5%
**Mobilisation**	Healthy work environment Collaboration	Staffing level	${\mathrm{Xbn}}_{1}^{M}$	90%	98%		92%
	Healthy work environment	Ratio of salary insurance hours	${\mathrm{Xbn}}_{2}^{M}$	7%	5,9%	13%	84.5%
	Healthy work environment Collaboration	Employee satisfaction rate	${\mathrm{Xbn}}_{3}^{M}$	70%	90%		70%
**Optimisation**	Innovation	Reference number change rate	${\mathrm{Xbn}}_{1}^{O}$	80%	90%		80%
	Innovation	Number of programs that have implemented the ‘compassion’ approach	${\mathrm{Xbn}}_{2}^{O}$	7 programs	9 programs	0 programs	77.8%

### Step 3: Indicator normalisation

The columns of Table 4 show the indicators measuring the objectives (and therefore sub-dimensions) selected in previous step, their notations, their values before normalisation, their targets, their worst values, and their values after normalisation (obtained by using equation (2)).

Due to confidentiality reasons, all real values of the indicators have been modified. Note that indicators that are already expressed as percentages before normalisation and which are aimed to be maximised to reach the desired target have not been normalised. The worst value is set to zero for those indicators (e.g. Customer satisfaction rate, Bed occupancy rate, and Respect of access deadlines) ***.*** Moreover, indicators before normalisation meant to be minimised to reach their target have been converted into indicators to be maximisation after normalisation (e.g. Complaint rate and Ratio of salary insurance hours).

### Steps 4 and 5: Indicator weighting and aggregation

Pairwise comparisons of normalised indicators within each QPM dimension were performed with the LN coordinator. The values obtained were entered in a VBA program that checks the consistency of the matrices and generates the weights following AHP algorithm (see Appendix A). The final results that represent the weights of the indicators are presented in Table 5.

**Table 5.** Calculating performance indexes for LN sub-service.

Dimension	Customer		Accessibility/Quality			Mobilisation			Optimisation	
$X_{i}^{\dim}$	$X_{1}^{C}$	$X_{2}^{C}$	$X_{1}^{A}$	$X_{2}^{A}$	$X_{3}^{A}$	$X_{1}^{M}$	$X_{2}^{M}$	$X_{3}^{M}$	$X_{1}^{O}$	$X_{2}^{O}$
$X_{i}^{\dim}$ (%)	75%	66.7%	85%	80%	82.5%	92%	84.5%	70%	80%	77.8%
$W_{i}^{\dim}$	067	0.33	0.44	0.44	0.11	0.25	0.50	0.25	0.33	0.67
Performance index ( ${\mathrm{PI}}_{\mathrm{LN}}^{\dim}$ )	72.2%		82.5%			82.3%			78.5%	

**Figure 10.**
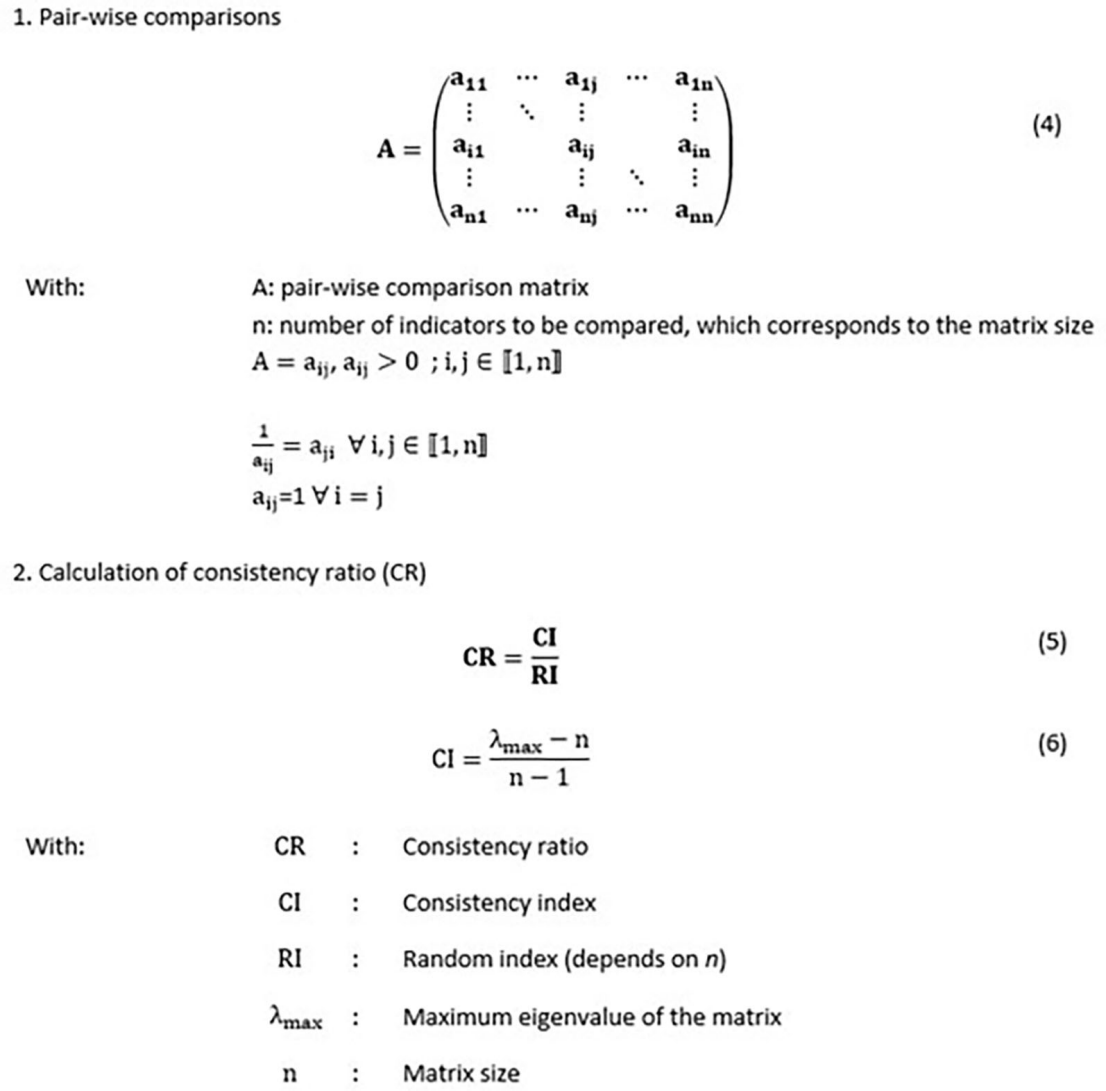
AHP calculation steps.

Step 5 enables us to calculate aggregated performance indexes for each dimension that can be used by the service managers or by higher hierarchy level departments (DP sub-program and DI-TSA-DP programs in our case) to evaluate how the service performs globally in each dimension of the QPM. For instance, if the global performance of a given dimension is not satisfying, the performance indicators within that dimension should be analysed in more detail to identify the problem and take actions to improve the situation. The results of calculating the four performance indexes $\left({\mathrm{PI}}_{\mathrm{LN}}^{C},{\mathrm{PI}}_{\mathrm{LN}}^{A},{\mathrm{PI}}_{\mathrm{LN}}^{M},{\mathrm{PI}}_{\mathrm{LN}}^{O}\right)$ of LN are given in Table 5. Each performance index corresponds to one dimension of the balanced-QPM. Again, the VBA program is used to automate these calculations following AHP algorithm. Figure 10 provides more details about the AHP calculation steps

.

## Discussion

The preliminary results of applying our approach to DI-TSA-DP program, and more specifically to LN service are promising. The starting point was the strategy map development, which is key for aligning DI-TSA-DP vision and strategy with the four dimensions/sub-dimensions of the QPM. This initial step helped to bring clarity and logic to the strategic objective determination process in relation to the QPM dimensions/sub-dimensions and performance measurement. Based on the strategy map, it was also possible to align LN service performance indicators with the four dimensions of the QPM through the strategic objectives and sub-dimensions selected. The strategy map plays a pivotal role in translating the program’s vision and strategy into strategic objectives and measurable indicators. Moreover, our approach contributes to balancing the four dimensions of the QPM by ensuring that a sufficient number of performance indicators measure each dimension. Performance indicators selection relied on a transparent and rigorous process that involved the participation of DI-TSA-DP and LN managers. To enable managers to evaluate the global performance of LN service, aggregating the performance indicators and calculating one performance index for each dimension by using an aggregative method (AHP) was appreciated by the managers.

CCSMTL (and DI-TSA-DP program) case study helped us to better understand the challenges and difficulties managers may face during strategy map construction and PMMS development/implementation processes. Regarding the strategy map, iterative meetings with DI-TSA-DP program managers were necessary to build the version presented in this work. More collaboration with the management team is required to improve this preliminary version (i.e. determining more precisely the strategic objectives and causal links between them). When using AHP technique, we identified a compensation effect between indicators that results from aggregating two (or more) indicators having opposite values, which is not desired. For instance, in our case study, within Mobilisation dimension, the normalised indicator ‘Staffing level’ ( $X_{1}^{M}$ ) has a value of 92% while the normalised indicator ‘Employee satisfaction rates ( $X_{13}^{M}$ ) has a value of 70%. Their aggregation together with the normalised indicator ‘Ratio of salary insurance hours’ ( $X_{2}^{M}$ ) (value of 84.5%), leads to a performance index with a value of 82,3% ( ${\mathrm{PI}}_{\mathrm{LN}}^{M}$ ) ( Table 5). It is easy to see that the high value of ‘Staffing level indicator’ compensates the lower value of ‘Employee satisfaction rate’ indicator. This result also depends on the weights assigned to the three indicators (0.25, 0.25, and 0.50, respectively in our case). We also observed that choosing targets for the indicators was not an easy task for managers. In fact, most existing indicators are associated with organisational targets that are set by strategic management. Due of this, it is challenging for managers to select reasonable targets for new indicators.

In addition, deep discussions took place between managers during the processes of comparing indicators to each other to agree on common preferences. This shows the difficulty of making common pair-wise comparisons and deriving the right weights, and therefore the right performance index values. This aspect was also observed in Boukherroub *et al.* (2017). It deserves to be addressed in future work, for instance, by considering group-decision-making techniques in the weighting process or by discussing this issue at the strategic management to set clear guidelines to support managers at different hierarchical levels in defining the weights that best reflect the vision of the organisation. Dependencies among aggregated indicators might also occur. As an example, in psychosocial services, waiting time and customer service time evolve in opposite directions as an improvement in one indicator decreases the other and vice versa (i.e. when a social worker spends more time with his/her current customers, new customers assigned to him/her will wait longer before they can be met) ( Boukherroub *et al.*, 2022). Therefore, methods that address this issue are also required. Typically, the ANP technique (Analytic Network Process), which is a generalisation of AHP, is appropriate for this situation. BWM (Best Worst Method) ( Singh & Singh, 2020) and MACBETH (Measuring Attractiveness by a Categorical Based Evaluation Technique) ( Bana e Costa & Vansnick, 1994) are other possibilities.

## Conclusions and research perspectives

The CCSMTL has developed a multi-dimensional performance model (Quality-Performance Model - QPM), however, its implementation across the organisation is very challenging. This study proposes an approach that contributes to addressing this issue. First, our proposed strategy map supports managers in identifying indicators that are aligned with their strategic objectives and vision. Second, the proposed PMMS helps them focus on all four dimensions of the QPM by explicitly linking performance indicators to each dimension. This, notably, supports proposing performance indicators within the customer dimension, which contributes to promoting customer-focussed culture. This would also allow departments to concretely measure their performance and compare themselves in a way that fosters competitiveness and anchors performance-oriented practices in the organisation. Third, the aggregated performance measures (performance indexes) that we propose to calculate based on AHP method will help managers at higher hierarchy levels to have a better visibility and understanding of the performance of operational teams, and make better decisions to support their improvement. Finally, our approach would support standardising performance management practices within the organisation. Our work contributes to the theory and practice by proposing a performance management and measurement approach adapted to large healthcare organisations, which is has been reported in practice and in the literature as a very challenging problem ( Moisan, 2019).

This study has some limitations. Strategy maps should be built for all programs of the CCSMTL prior to indicator selection, validation, information aggregation, etc. to align the measured performance with strategic objectives. Brainstorming workshops should be carried out with managers and coordinators to establish more precisely their objectives and causal links between these objectives. More work is also required to design additional indicators. In particular, means of measuring and monitoring indicators over time need to be developed. Since AHP does not take into consideration potential interdependences between indicators, ANP, BWM, and MACBETH techniques could be explored. Undesired compensation effects might also occur in the aggregation process. Finally, it is not recommended to limit the use of performance indexes alone for monitoring performance. Operational indicators related for instance to emergency services are very important to monitor at the highest management level.

The following is a testimonial from the deputy director of organisational performance at the CCSMTL: *“As part of this project, several working sessions were held with stakeholders [CCSMTL managers]. These structured interviews enabled the research team to well capture the QPM and then develop their approach accordingly. The data [performance indicators] organisation structure is very relevant in the sense that it addresses both the dimensions and the sub-dimensions of the QPM, and it is declined according to the hierarchical administrative structure of the CCSMTL, thus matching with our approach of continuous improvement, which aims to dynamise the information cascade-escalation process through all layers of the organisation. Finally, the rigorous process of validating and weighting the indicators was carried out with the stakeholders, thus ensuring better reliability of the indicators developed, which will optimise their use to eventually support decision-making.”*

## Data Availability

No data are associated with this article.
