# Towards an Integrated Performance Management and Measurement System for healthcare organisations: a case study in Montreal

**DOI:** 10.12688/f1000research.138430.2

**Published:** 2024-12-09

**Authors:** Anes Ben Fradj, Neila El Asli, Tasseda Boukherroub, Claude Olivier

**Affiliations:** 1 Numérix Laboratory, École de technologie superieure, Montreal, Québec, Canada; 2 Systems Engineering Department, École de technologie superieure, Montreal, Québec, Canada; 3 Interuniversity Research Centre on Enterprise Networks, Logistics and Transportation, Montreal, Québec, Canada; 4 Industrial Engineering Department, Université de Quebec à Trois Rivieres, Quebec, Canada

**Keywords:** AHP, BSC, performance management, healthcare

## Abstract

This study proposes an approach for developing or improving performance management and measurement systems (PMMSs) for healthcare organisations. First, data is collected to analyse and understand the current organisation’s performance management system. Second, the SWOT (Strengths, Weaknesses, Opportunities, Threats) method is used to identify the main aspects of the performance management system to be improved. Third, based on the scientific literature and SWOT analysis, BSC principles are integrated to this performance management system to better align the organisation’s performance objectives and indicators with its strategy. Finally, we develop a performance indicator structure and select indicators to be used as well as how these indicators could be integrated and shared with higher hierarchical levels in the organisation by using AHP (Analytic Hierarchy Process). Our approach is applied to the CIUSSS du Centre-Sud-de-l’île-de-Montreal (CCSMTL), a large healthcare network, in the province of Québec, Canada.

## Introduction

In the province of Québec, Canada, public health and social services are provided by networks of institutions called CI (U)SSSs (integrated (University) centres of healthcare and social services), which fall under the responsibility of the Ministry of Healthcare and Social Services (Mimistère de la Santé et des Services Sociaux referred to as MSSS). Their main mission is to provide healthcare and social services to the population while ensuring accessibility, quality, safety, and efficiency. This study focusses on the CIUSSS serving the South-Centre of Montreal region (CIUSSS du Centre-Sud-de-l’île-de-Montréal referred to as CCSMTL). It provides 28 different healthcare services to a population of 311,000 people, it owns 150 facilities and employs more than 21,300 people, including 56 senior managers and nearly 800 physicians. It manages a budget of more than $1.7 billion Canadian dollars ( Annual Report 2019-2020, CIUSSS). When the CCSMTL was created in 2015, it had to develop a reference framework for its performance management and to define a vision regarding its organisational performance. A *Management and Accountability Agreement*, involving the MSSS and the CCSMTL management, was introduced by the MSSS, with the obligation to implement a visual management system as a means for performance management and decision-making. Therefore, the CCSMTL has built its Quality-Performance Model (referred to as QPM), which encompasses four dimensions of performance; customer, accessibility/quality, mobilisation, and optimisation. It has also implemented its visual management system. However, QPM deployment is very challenging:

1) It must be implemented at different decision-making levels, organisational structures (programs, sub-programs, services, teams, etc.), and territories. For instance, performance indicators may differ greatly from one service to another or from one territory to another.
2) Some performance aspects such as customer and mobilisation dimensions are not sufficiently or well measured, which leads to an unbalanced performance management system.
3) CCSMTL managers need to measure the overall performance of a given service or program, through integrated information. Currently, such information is not available nor easy to obtain.

This study contributes to addressing the aforementioned issues. More precisely, it proposes a four-phases based approach to support CCSMTL managers to improve and transform the QPM into a more balanced and effective performance management and measurement system (PMMS). Even though recent studies in the literature discuss performance management systems in healthcare organisations and recognise both the importance and the complexity of measuring performance, most of them are descriptive and do not propose PMMSs capable of effectively structuring and measuring the performance of healthcare organisations. Our work contributes to the theory and practice by proposing a comprehensive and quantitative performance management and measurement approach adapted to large healthcare organisations such as the CCSMTL. It can be used by theoreticians and practitioners as roadmap to develop or improve healthcare organisations’ PMMSs. The remainder of this paper is organised as follows: the next section presents the QPM in more detail. After that, we present our literature review and then describe our approach. Next, we present the results of applying our approach to the CCSMTL case study. We also discuss the preliminary results. Finally, we conclude and present some research perspectives.

## The quality-performance model

**Figure 1.**
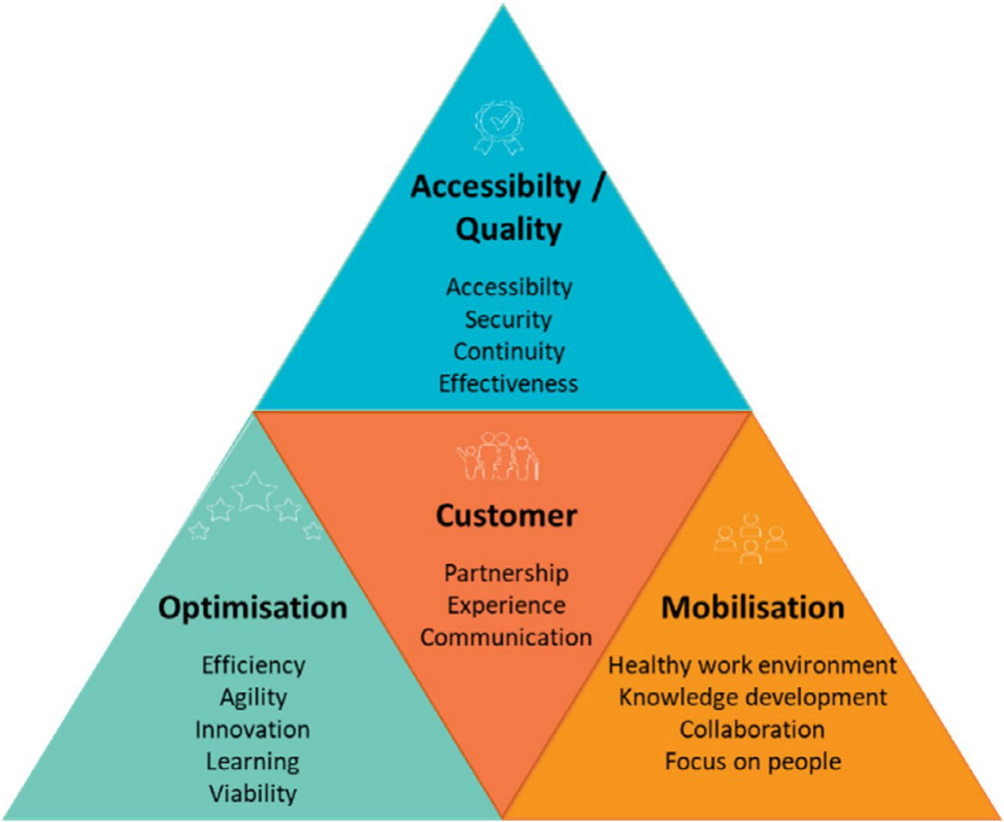
Quality-Performance Model (QPM) ( Couillard, 2018).

The QPM (see Figure 1) is based on the MSSS strategic plan (2019-2023 period) and framework for assessing the performance of the public healthcare and social services system ( MSSS, 2012), Accreditation Canada’s quality management framework ( Agrément-Canada, 2014), and PLANTREE’s person-centred approach ( Cosgrove, 1994). The QPM encompasses four dimensions of performance: customer, accessibility/quality, mobilisation, and optimisation. Each dimension presents an aspect of the CCSMTL performance and encompasses a number of sub-dimensions enriched by the perspectives of the customers and the community (see Table 1).

**Table 1.** QPM dimensions and sub-dimensions enriched by the customer and community perspective ( Couillard, 2018).

Dimension	Sub-Dimension	Definition
**Customer**	**Partnership**	Involve myself and my relatives as partners in the care and services you provide
	**Experience**	Make sure you have a human relationship with me so that I have a positive experience
	**Communication**	Listen to me, inform me of my rights and give me complete information in accessible language
**Accessibility / Quality**	**Accessibility**	Welcome me with an open mind and provide me in a timely fashion with appropriate care and services
	**Security (safety)**	Ensure my safety
	**Effectiveness**	Do what it takes to provide me with the best care and services
	**Continuity**	Coordinate my care and services throughout my trajectory
**Optimisation**	**Efficiency**	Avoid waste when using your resources
	**Viability**	Consider all perspectives to meet my present and future needs
	**Agility**	Be proactive and responsive to my expectations, values, and rights
	**Innovation**	Dare to introduce new ways to offer care and services
	**Learning**	Learn from experience how to do better
**Mobilisation**	**Healthy work environment**	Take care of those who care for me and humanise the physical environment
	**Focus on people**	Work with community partners who can help us
	**Knowledge development**	Support those who care for me to maintain and develop their knowledge
	**Collaboration**	Talk to each other and work together

Accessibility/quality and customer are the two main dimensions of the QPM. They are presented in the form of a compass needle that points in the right direction to achieve better performance. Positioned at the centre of the model, customer dimension focusses on customer satisfaction regarding services and care provided. It presents three sub-dimensions: partnership, experience, and communication. Customers refer to people who receive a service or care and their relatives, as well as the community and the population in general. Accessibility/quality is the organisation’s ability to meet safely the needs and expectations of customers and ensure accessible and continuous services. It presents three sub-dimensions; accessibility, safety, and relevance/effectiveness of services. Mobilisation is the use of skills and talents of all people working in the organisation as well as fostering personal achievement and commitment to fulfil collectively the organisation’s mission. It encompasses four sub-dimensions, efficiency, agility, innovation, learning, and sustainability. Finally, optimisation is the search for continuous improvement in order to use efficiently available resources and provide services that are adapted to customer needs. Its sub-dimensions include healthy work environment, knowledge development, collaboration, and focus on people.

The CCSMTL also implemented its visual management system formed by a network of control rooms at different decision levels of the organisation. Put simply, a control room is a dedicated space where managers and employees meet regularly to acquire information about current situation and initiate discussions to improve future performance ( Lagacé et Landry, 2016). At the highest organisational level, we find the strategic room, which is used by the CEO and senior managers. Data is used to evaluate the organisational performance, monitor current strategy, and support strategic decision-making (e.g. prioritising major projects). Tactical rooms are deployed in the different programs and sub-programs. Managers discuss their departments’ performance and decisions to be made at their level. Some of these decisions flow-up to the strategic room for validation while others flow-down to ‘intermediate’ tactical rooms or operational control rooms to be executed, in a cascade-escalation mechanism ( MSSS, 2015). Some tactical rooms referred to as “intermediate tactical rooms” might play an intermediate role between tactical and operational levels. Operational control rooms (or visual stations) ensure day-to-day operations management ( MSSS, 2015). The QPM is aimed at being implemented in all CCSMTL control room networks to support performance management.

As mentioned earlier, QPM deployment is very challenging for the CCSMTL. First, it must be implemented at different decision-making levels, organisational structures, and territories. However, performance indicators may differ greatly from one service to another or from one territory to another. Second, some performance aspects such as customer and mobilisation dimensions are not sufficiently or well measured, which leads to an unbalanced performance management system. Finally, CCSMTL managers need to measure the overall performance of a given service or program, through integrated information. Currently, such information is not available nor easy to obtain.

## Literature review

Measuring the performance of healthcare organisations might be very challenging ( Ferreira et al., 2009; Fache, 2016; Solomon, 2013) notably in complex organisations. Among recent studies that reported on performance management in healthcare organisations, we can cite Moisan (2019) who focused on the CISSS of Gaspésie region (province of Québec). This study highlighted the experience of this institution in deploying its performance management system and described how it contributed to improve the client trajectory performance in the context of social services dedicated to youth. Fache et al. (2016) investigated the role of the Department of Health in Quebec which faces the situation of *"indicator chaos".* To the authors, this *chaos* is at the quantitative level (too many indicators) and at the qualitative level (different measures for the same indicator), and consequently, this situation complicates effective organisational management and evaluation. Ferreira and Otely (2009) introduced the performance management system framework as a research tool aimed at providing a more comprehensive description of the structure and functionality of performance management systems. Their framework was developed based on a synthesis of relevant literature and insights gathered from their observations and experience. Patient-reported outcome and experience measures (PROMs and PREMs) for system performance measurement is considered as a way to enhance and promote the shift of healthcare systems towards a more coordinated, integrated and people-centred care. This approach has been the focus of recent studies such as Bull and Callander (2022) and Nuti *et al*. (2017) who discussed the importance of using patient-reported measures as well as indicators based on administrative data to evaluate cross-sectional healthcare services in a multidimensional healthcare performance system. Bull and Callander (2022) discussed the different performance measurement programs employed across England, the US and Australia, and suggested key recommendations for advancing PROM and PREM programs internationally.

A performance measurement system could be seen as the process of assessing the organisation’s progress in achieving its goals ( Kairu *et al*., 2013). It contains financial and non-financial measures ( Okwo & Marire, 2012). It should enable the correct deployment of the strategic and tactical objectives and provide a structured framework enabling the relevant feedback of information to the appropriate levels to facilitate decision-making and control ( Bititci *et al.*, 1997). Botton *et al.* (2012) classified performance measurement systems according to three categories of approaches: strategy-oriented (BSC ( Kaplan & Norton, 1996), performance prism ( Neely *et al*., 2002)), hierarchical (performance pyramid), and process-based (macro-process model ( Brown, 2008)). The QPM can be considered as strategy-oriented. Due to its multi-dimensional and strategy-focussed aspects, it is consistent with the BSC model. In their recent systematic review, Amer *et al*. (2021) provided managers and policymakers with evidence to support using BSC in the healthcare sector. They conclude that BSC implementation demonstrated positive outcomes for patient satisfaction and financial performance. In their systematic literature review, Betto *et al*. (2022) attempted to understand the evolution of BSC in healthcare and their results revealed that the majority of studies focussed mainly on the BSC design process, rather than BSC implementation, use, or review. To Bohm *et al*. (2021), the composition of BSC development teams in healthcare should consider including patients as well as their families where appropriate and select highly achievable and valid performance measures aligned with the organisational strategy.

Developed in 1992 by Kaplan and Norton, the BSC is the result of a re-examination of performance measurement systems that usually give too much importance to the financial aspect. It is designed to measure four perspectives: finance (measures financial performance and the efficient use of financial resources), customer (concerns the organisation’s impact on the market in terms of customer satisfaction), internal processes (focusses on the efficiency and quality of internal processes) and learning & growth (concerns the culture and perspectives of the organisation, human resources, and information system). The term “balanced” underlines the concept of balance between all four axes of the model ( Kaplan & Norton, 1992). In BSC model, we distinguish leading indicators, which measure processes, and lagging indicators that measure outcomes. Causality is one of the most important aspects in the implementation of BSC: there is an implicit causal relationship between BSC perspectives and lag-lead indicators ( Porporato, Tsasis, et Vinuesa, 2017). To this end, a visual map (or strategy map) is built to show the objectives and causal links needed to execute a strategy. Scholey (2005) provides a well-structured six-step based process for creating a strategy map. Kaplan and Norton stressed the need to measure causal relationships, by using a complementary analytical approach ( Barnabè *et al*., 2012) even though, it is not easy to analytically validate all causal relationships ( Lorino, 2001; Schrage & Kiron, 2018). This is also the case for healthcare ( Porporato *et al*., 2017).

**Figure 2.**
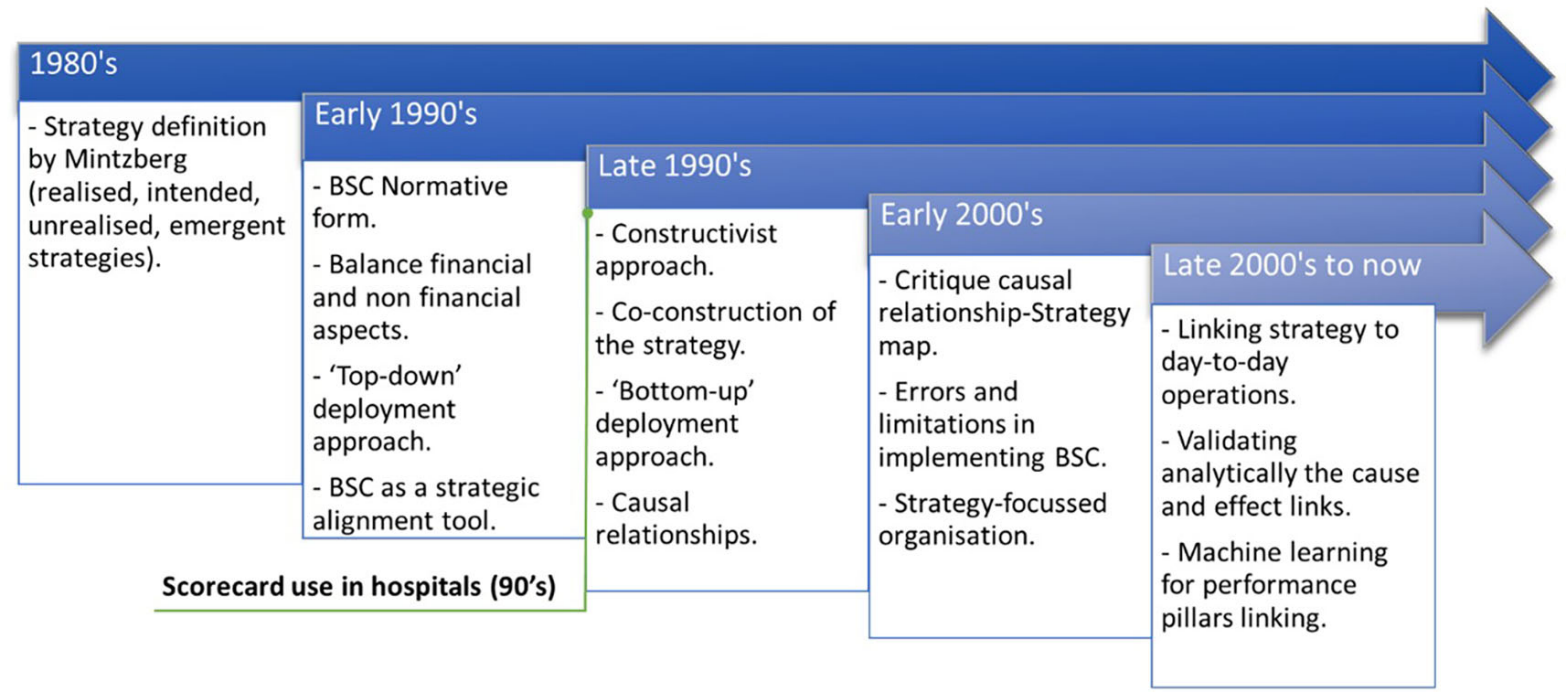
BSC evolution over time and application in healthcare.

Canada leads in hospital performance measurement and historically, it is among the first countries to develop scorecards in healthcare ( Porporato *et al*., 2017). In Toronto, Ontario, Women’s College Hospital started using a scorecard based on key objectives in the 1990’s ( Porporato *et al*., 2017). Since then, Ontario hospitals have gained great experience in designing and using scorecards. Chan (2009) presented the evolution of healthcare systems in implementing BSC and strategy maps in Ontario. From 1992 to date, BSC has evolved from a strategic alignment tool (normative form) to a strategy construction tool. The normative version proposes a “top-down” deployment approach to operationalise the organisational strategy. Studies have shown that BSC can contribute to the emergence of a new strategy, and it would be better to opt for a “bottom-up” approach, where strategic objectives would be the result of collaborative management ( Choffel & Meyssonnier, 2005; Ittner & Larcker, 2003; Norreklit, 2000; Simons, 1994). In addition, the learning & growth axis should aim to shape the organisation’s culture. Kaplan (2020) presented the case of merging St. Mary’s Hospital and Duluth Clinic (Minnesota, US) who co-created a new strategy, a strategy map and a common BSC. Montalan and Vincent (2011) proposed an approach for the co-construction of the strategy of a French hospital. They developed and validated a strategy map by a multidisciplinary teamwork following a bottom-up approach. Figure 2 shows the evolution of BSC over time and application in healthcare.

In order to measure the performance while considering the different dimensions of the BSC (healthcare and other sectors), some studies in the literature combined the BSC with multi-criteria decision-making techniques (MCDM). Most of these studies used the well-known Analytic Hierarchy (or Network) Process (AHP/ANP) ( Anjomshoae *et al*., 2019; Chan, 2006; Modak *et al.*, 2019; Regragui *et al.*, 2018; Yaghoobi & Haddadi, 2016). Chan (2006) combined AHP and BSC to identify global metrics and compare performance across Canadian healthcare organisations. Regragui *et al.* (2018) also combined BSC and AHP to develop a framework for assessing performance in hospitals. By using AHP, the authors weighted different performance perspectives and indicators, leading to a ranking of these indicators. In other studies, AHP has been successfully used to consolidate, prioritise, and weight performance indicators ( Bentes *et al*., 2012; Hossain *et al.*, 2019; Yaghoobi & Haddadi, 2016). For more on AHP applications in healthcare, see Hummel and IJzerman (2011). Recently, Daneshmand *et al*. (2022) developed a BSC-AHP based model for assessing the health system of the Social Security Organisation in Iran. They conclude that, the most important performance dimensions are internal processes and social obligations. Table 2 presents most recent BSC applications in healthcare including studies that combined BSC and AHP. Our proposed approach is inspired by the work of Regragui *et al.* (2018).

**Table 2.** Examples of most recent BSC applications in healthcare.

Country	Perspective	Reference
Australia	Validate a non-profit version of the BSC	( Soysa *et al.*, 2019)
	Integrating an environmental dimension with BSC	( Khalid *et al.*, 2019)
Brazil	Performance Management - survey on performance management practices in Brazil	( Portulhak *et al.*, 2017)
Canada	Performance measurement - implementation of a bottom-up approach	( Backman *et al.*, 2016)
	Integrating AHP to hospital scorecards in performance assessment	( Chan, 2006)
	Information management - key indicators - strategic alignment	( Nippak *et al.*, 2016)
	Causal Relationships	( Porporato *et al.*, 2017)
	Queue performance evaluation	( Breton *et al.*, 2017)
China	Establishing an Indicator System using the BSC	( Gao *et al.*, 2018)
Ethiopia	Performance measurement - development of a scoring methodology	( Teklehaimanot *et al.*, 2016)
Germany	Prioritise strategies	( Dekrita *et al.*, 2019)
India	Dynamic Balanced scorecard and its connectivity with Corporate Social Responsibility	( Ghosh & Singh, 2021)
Iran	Performance measurement system for the Social Security Organisation's health system.	( Daneshmand *et al.*, 2022)
Morocco	Conceptual framework for evaluating performance	( Regragui *et al.*, 2018)
Saudi Arabia	Cloud computing - Strategy map	( Alharbi *et al.*, 2016)
UK	Success factors for performance management	( Moullin, 2017)
USA	Accreditation in hospitals (ISO9001)	( Ritchie *et al.*, 2019)
	Merging health care organisations	( Kaplan, 2020)
Vietnam	Performance evaluation	( Pham *et al.*, 2020)

## Methods

**Figure 3.**
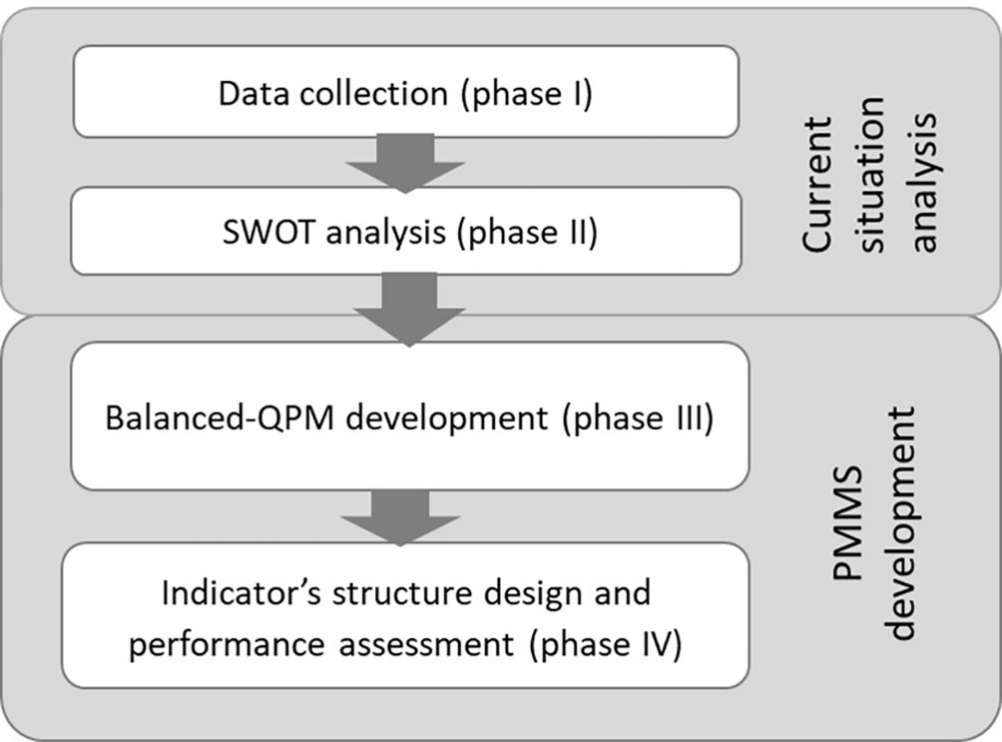
Proposed approach.

Our approach encompasses two parts as illustrated in Figure 3. The first part (Phases I and II) aims at describing and analysing the current situation while the second part aims at developing or improving a PMMS (Phases II and IV).

### Current situation analysis (Phases I and II)

The data collection (phase I) began in May 2019 (the study started in January 2019). Collected data was used to gain a more in-depth understanding of CCSMTL internal processes and current practices and progress regarding the implementation and use of its visual management system and QPM (deployment level, indicators, implementing obstacles and issues, etc.). To this end, three methods were used: review of documents, field observations, and structured interviews (see Table 3).

**Table 3.** Description of the used methods for phase I.

Data collection methods	Description
Document review	MSSS’s Law on healthcare and social services, Health Standards Organization (HSO) standards ( HSO, 2021), Accreditation Canada ( Agrément-Canada, 2014), Ministerial reference framework for evaluating performance in public healthcare and social services systems ( MSSS, 2012), MSSS strategic plan 2019-2023, CCSMTL annual reports ( CCSMTL, 2016), and PLANETREE documents (e.g. Cosgrove, 1994).
Observations	1-Participation in the QPM Coordinating Committee and the QPM Advisory Committee meetings. 2- Participation in a performance improvement workshop. 3-Visiting several control rooms of the CCSMTL at different levels of the organisation: the strategic room, five tactical and intermediate rooms, and 10 operational rooms. 4-Visiting two hospitals and two rehabilitation centres.
Structured interview	12 structured interviews involving 13 managers (Directors, assistant managers, executive advisors, department heads) and four Programs and departments (i.e. different decision-making levels and services) between May 2019 and September 2021. The following are examples of questions asked during the interviews: - Are the strategic objectives targeted by the implementation of the QPM? - What are the practices put in place for implementing the QPM? - How does the CCSMTL measure performance and quality in the organisation? - What are the difficulties met during your performance measurement process?

The Coordinating Committee of the CCSMTL is composed of a limited number of managers from the Quality, Evaluation, Performance and Ethics department. It aims at implementing the QPM within the CCSMTL, in accordance with the MSSS strategic plan. Periodically, the Coordinating Committee invites the heads of all CCSMTL departments to discuss decisions to be made related to the QPM and collect their feedback. This enlarged group forms the QPM Advisory Committee. The objective of the interviews (with a duration of 1 to 2 hours) was to validate and consolidate our understanding of the performance management and measurement methods and tools being used and the progress in implementing the QPM and visual management system. The structured interview was used ( DiCicco‐Bloom and Crabtree, 2006) the interviewer asks several structured questions, and the interviewee responds freely to the questions. The participants were selected in collaboration with the deputy director of organisational performance at the CCSMTL, who is responsible for QPM implementation. When required, a second meeting was scheduled with the same participant(s). For more details, the reader may refer to Ben Fradj *et al.* (2020).

Next (Phase II), the SWOT (Strengths, Weaknesses, Opportunities, Threats) method was used to structure and analyse the collected data. SWOT analysis is a popular tool from which strategies can be developed and which improves organisational performance ( Pickton and Wright, 1998). It helps to clarify the strengths to be maintained, the weaknesses to be addressed, the opportunities to benefit from, and the constraints to be handled. In our case, we used the SWOT to identify the strengths and weaknesses related to the current implementation of the QPM model. Once the data was collected, the research team brainstormed to cocreate the preliminary version of the SWOT matrix. Next, four discussion meetings have been organised with three different managers from the CCSMTL to validate and complement the final version of the SWOT (see Table 4 in the Results Section).

### PMMS Development (Phases III and IV)

***Balanced-QPM and strategy map development (Phase III)***

Based on the results of our SWOT analysis and inspired from the literature, we use BSC principles to create a more balanced QPM. In other words, we use the BSC in order to address the weaknesses of the QPM model identified in our SWOT analysis. Indeed, by comparing BSC and QPM models, we identified similarities and complementary aspects. First, there is a strong similarity between the dimensions of performance of the QPM and the axes of the BSC, in the way that they express equivalent pillars of performance (customer, internal processes, resource management, etc.). Second, both models enable defining performance indicators and creating a measurement system. Third, the QPM and the BSC provide a basis for stakeholders to share and discuss strategic objectives. Finally, the BSC is often recommended in the healthcare sector as mentioned in the literature review section.

First, the axes of the BSC, i.e., the version adapted for non-profit organisations by Kaplan, and the dimensions of the QPM are compared to each other. For example, BSC’s customer perspective corresponds to the customer dimension of the QPM. In the CCSMTL context, achieving the goals of this dimension reflects the organisation’s main mission and therefore would take the first position in the Balanced-QPM. Similarly, BSC’s internal process perspective is equivalent to the accessibility/quality dimension in the QPM. We recall that accessibility/quality refers to the ability to safely meet customer needs and expectations by providing accessible and continuous services. This dimension would take the second position in the Balanced-QPM. The positioning of customer and accessibility/quality dimensions is consistent with the CCSMTL vision which considers the two dimensions as the “True North” of the organisation. Second, based on the causal relationship between the BSC axes, we identify the causes and effect links specific to the dimensions of the QPM model. This aspect was absent in the intitial QPM. For instance, the optimisation dimension has direct impact on the accessibility/quality dimension, which in turn has a direct effect on the customer dimension. The result of these two processes (positioning the QPM dimensions and identifying causal relationships between them) leads to what call the Balanced-QMP (see Figure 5 in the Results Section).

To create the CCSMTL’s strategy map, the causal links between the Balanced-QPM dimensions are further analysed. We were inspired by the six-step guidelines suggested by Scholey (2005). To the author, this process leads to the creation of a comprehensive strategy map, ensuring a clear and communicable strategy throughout the organization. It consists in 1) identifying the primary objective, 2) selecting the value proposition, 3) determining general financial strategies to follow, 4) establishing customer-focused strategies, 5) aligning internal processes to support strategy execution, and finally 6) implementing necessary skills, capabilities, and employee programs to achieve the strategy.

For the CCSMTL, the primary objective is to satisfy its customers by providing general and specialised healthcare and social services while insuring accessibility, security, efficiency and quality. An example of a target to achieve for this primary objective would be to increase the access delay compliance rate to 95% within one year. For the value proposition selection, we relied on the study by Treacy and Wiersema (2007), who identified three value propositions; operational excellence, product excellence and customer relationship. Since the CCSMTL follows a customer-oriented approach, its value proposal focusses on customer relationship. The remaining step are based on the QPM sub-dimension definitions enriched by the customer perspective (see Table 1) that could be further expanded by the objectives set by the department managers, in relation to their specific mission. In addition, rigorous criteria for selecting relevant objectives such as specificity, validity, sustainability, reliability, and utility could be used to support this process. The resulting strategy map is shown in Figure 6 in the Results Section.

### Indicator structure design and performance assessment (Phase IV)

This phase aims at providing relevant indicators measuring performance following the objectives specified in the strategy map in each performance dimension.

### Step 1: Indicator system structure

We propose to create a structured system that provides indicators as well as performance indexes for each performance dimension at each hierarchical level of the CCSMTL. This general structure is based on the CCSMTL organisational chart and its visual management system. To provide and monitor the performance indicators, scorecards presenting the four dimensions (and their indicators) can be used at each department and hierarchical level, forming a network of scorecards (information is presented and discussed in the control rooms during regular meeting. This proposal is inspired from the study of Voyer ( Voyer & Voyer, 1999) who developed a network of interrelated dashboards used at different levels within an organisation. Béland and Abran (2004) also used this concept (see Figure 7 in the Results Section).

### Step 2: Indicator identification and selection

From the established strategy map, performance indicators are selected for each dimension following strategic objectives that are relevant to the department/service. Existing indicators should be reviewed first. As suggested by Bull and Callander (2022) and Nuti *et al.* (2017) patient-reported measures and indicators based on administrative data can be used. Existing indicators and new ones (selected based on the literature or on recognized standards) should be elected according to criteria ensuring specificity, validity, sustainability, reliability, utility, etc. Such criteria are well documented in the literature for selecting relevant performance objectives and indicators. At the end of this step, each department will have a set of indicators for each dimension of the Balanced-QPM called “before normalisation indicators”, noted as Xbn in the following paragraphs.

### Step 3: Indicator normalisation

The “before normalisation indicators” Xbn may have different units of measurement and different magnitudes in healthcare contexts. In some cases, the measurement unit is expressed in monetary units (e.g. expenses), and in others, in time units (e.g. hours) such as waiting time. In other cases, there is no measurement unit (e.g. number of services provided). In addition, the same indicator may have distinct targets in different departments. For example, waiting time target in an emergency department is significantly less compared to psychosocial services. Therefore, it might be difficult to compare them to each other based on their absolute values. Due to this, normalising the indicators is very helpful. Normalisation techniques for processing data have been used by many researchers in different fields. We refer the reader to Singh and Singh (2020) for more on normalisation. We selected the Min-Max normalisation technique ( Singh & Singh, 2020), mainly because minimum and maximum limits can be easily distinguished in available data provided by the CCSMTL. Following this technique, data is usually rescaled within the range 0 to 1 or (-1) to 1. The general equation is given as follows:

$$X_{\mathrm{norm}}=(\text{Nmin}-\text{Nmax})\frac{X_{\mathrm{bn}}-\min}{\max-\min}+\text{Nmin}$$ (1)

With:

$X_{\mathrm{norm}}$ : Normalised value of the indicator

$X_{\mathrm{bn}}$ : Indicator value before normalisation

min : Minimum value in the data

max : Maximum value

Nmin;Nmax : Normalised Min and Max

For the CCSMTL context $X_{\mathrm{norm}}$ is expressed as a percentage ranging from 0% to 100%, therefore Nmin et Nmax values are 0% and 100%, respectively. Given that in some cases the maximum value of an indicator is the undesirable value while the minimum value is the desired value (e.g. the maximum value for waiting time is non-desired while the minimum value is desired), we set the best value in the data set as max parameter (which is not necessarily the highest value) and the worst value in the data as min (which is not necessarily the lowest value) before normalisation. Outliers must be eliminated before choosing the best and worst values. To link a given indicator to a desired objective and to ensure its continuous improvement, max value corresponds to the indicator target. For each performance dimension, there is a set of associated indicators. Each indicator takes a numerical index i in the set and each indicator has an index *dim* referring to the first letter of the dimension to which it belongs (e.g. C for Customer). Indicators before normalisation take the index *bn*, referring to “before normalisation”. Normalised indicators in the original formula ( Equation (1)) $X_{\mathrm{norm}}$ become $X_{i}^{\dim}$ in the new formula adapted to the CCSMTL ( Equation (2)). We remove the index *norm* for the sake of simplicity.

$$X_{i}^{\dim}=(100)\times \frac{{\mathrm{Xbn}}_{i}^{\dim}-\min_{i}}{{\text{target}}_{i}-\min_{i}}$$ (2)

Each department/service at different hierarchical levels calculates its normalised indicators ( $X_{i}^{C}$ , $\;X_{i}^{A}$ , $\;X_{i}^{M}$ , $\;X_{i}^{O}$ ). These are the inputs of the fourth step.

### Step 4: Indicator weighting

**Figure 4.**
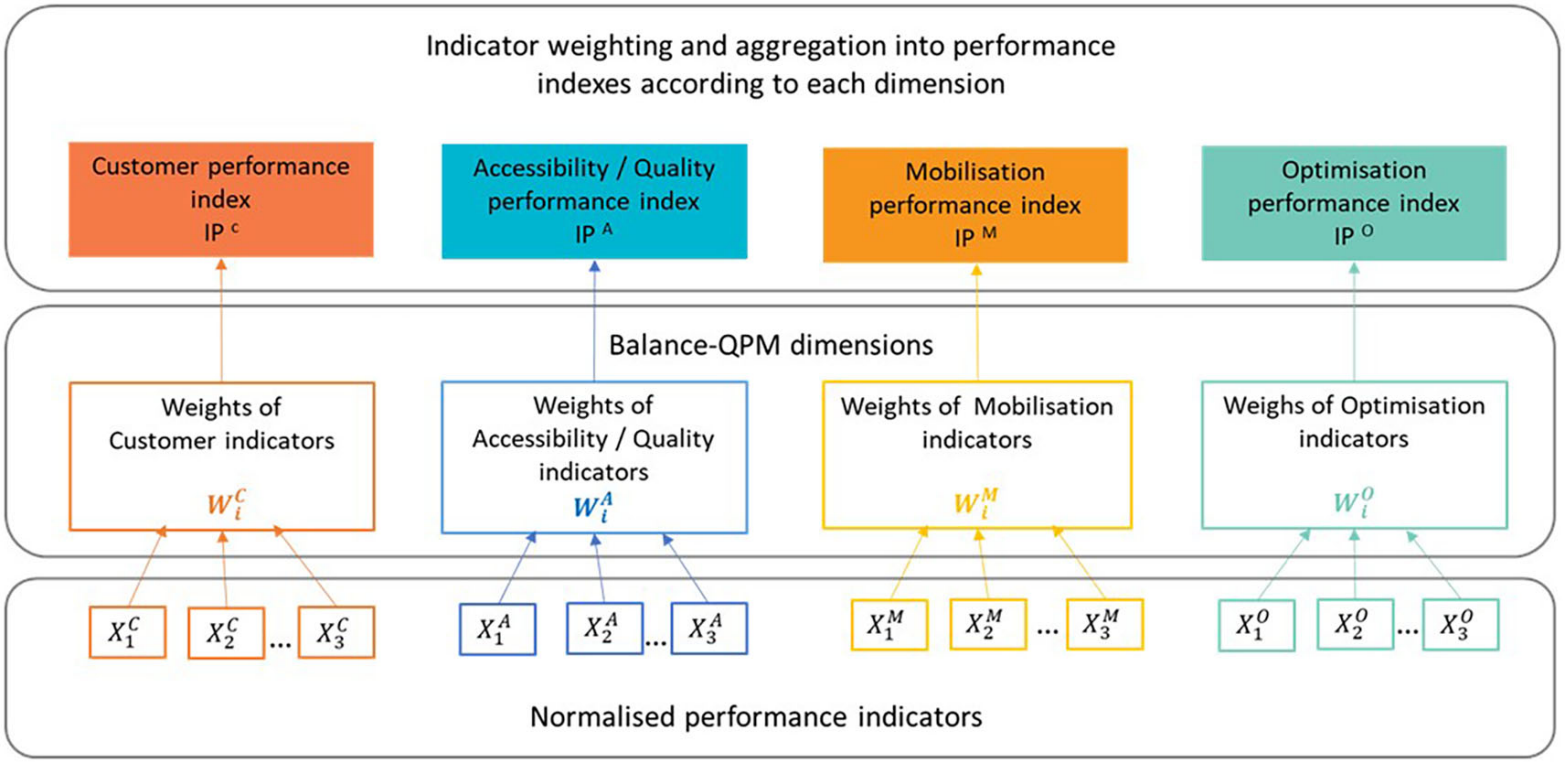
AHP adaptation for performance measurement for the CCSMTL case.

Step 4 aims at weighting the normalised indicators of the same dimension at the same hierarchical level. AHP technique is used. AHP is usually used to evaluate and rank a set of evaluated based on several criteria. In this study, there is no need to rank or choose an option, but rather weighting the indicators within each performance dimension and aggregating them to obtain performance indexes for each dimension for a given department (see Figure 4). We selected AHP due its simplicity ( Kumar *et al*., 2017) since it is very convenient for decision-makers who are not familiar with analytical tools. Moreover, it relies on pairwise comparisons, and according to Ishizaka and Labib ( Ishizaka & Labib, 2011), psychologists argue that it is easier to express an opinion based on two elements rather than on all elements simultaneously.

Criteria and indicators that are usually used in AHP, correspond to the four dimensions of the Balanced-QPM and to the normalised indicators $X_{i}^{\dim}$ , respectively. The normalised indicators are compared to each other by using pair-wise comparison matrices (one matrix for each dimension) according to their relative importance regarding the dimension to which they belong. These preferences are expressed by managers. A given element *a*_*ij*_ of a matrix A (see Equation (4) below) corresponds to the relative importance of indicator *i* (in the row) compared to indicator *j* (in the column). The preference values range from one to five, which is adapted from the preference scale proposed by Saaty (2005). For example, *a*_*ij*_ = 1 means that the indicators are equally important and *a*_*ij*_ = 5 that indicator *i* is extremely important compared to indicator *j*. The weights of the performance indicators are calculated by using a specific algorithm described in detail by Brunelli (2014). All AHP calculation steps are presented in the next paragraphs. Comparisons make sense only if the pair-wise comparison matrices are coherent or quasi-coherent. A consistency ratio (CR) is used to check this aspect. If CR is less than 10%, the matrix is consistent, otherwise, the pair-wise comparisons are repeated (see Equations (5) and ( 6) below). Once consistency is validated, the weights of the indicators, noted as $W_{i}^{\dim}\;$ ( $W_{i}^{C},W_{i}^{A},W_{i}^{M},W_{i}^{O})$ are obtained.

### Step 5: Indicator aggregation

Indicators within each dimension are aggregated (see Figure 8), and four performance indexes $\left(P^{C},\;P^{A},\;P^{M},\;P^{O}\right)$ are calculated (one index for each dimension) following Equation (3):

$$P^{\dim}=\sum_{i}^{n}W_{i}^{\dim}\times X_{i}^{\dim}$$ (3)

$P^{\dim}$ : Performance index of dimension *dim*

$W_{i}^{\dim}$ : the weight of indicator *i* within the performance dimension *dim*

$X_{i}^{\dim}$ : i *th* normalised indicator within performance dimension *dim*

Equations (4) to (6) provide more details about the AHP calculation steps.

$$A=(\begin{array}{ccccc} a_{11} & \cdots & a_{1j} & \cdots & a_{1n}\\ \vdots & \ddots & \vdots & \; & \vdots \\ a_{i1} & \; & a_{\mathrm{ij}} & \; & a_{\text{in}}\\ \vdots & \; & \vdots & \ddots & \vdots \\ a_{n1} & \cdots & a_{\mathrm{nj}} & \cdots & a_{\mathrm{nn}} \end{array})$$ (4)

With:

A: pair-wise comparison matrix

n: number of indicators to be compared, which corresponds to the matrix size

$A=a_{\mathrm{ij}}$ , $a_{\mathrm{ij}}>0$ ; i,j∈⟦1,n⟧

$\frac{1}{a_{\mathrm{ij}}}=a_{\mathrm{ji}}$ ∀i,j∈⟦1,n⟧

$a_{\mathrm{ij}}$ =1 ∀i=j

2. Calculation of consistency ratio (CR)

$$\mathrm{CR}=\frac{\mathrm{CI}}{\mathrm{RI}}$$ (5)

$$\mathrm{CI}=\frac{\lambda_{\max}-n}{n-1}$$ (6)

With:

CR: Consistency ratio

CI: Consistency index

RI: Random index (depends on *n*)

$\lambda_{\max}$ : Maximum eigenvalue of the matrix

n: Matrix size

## Results

**Figure 5.**
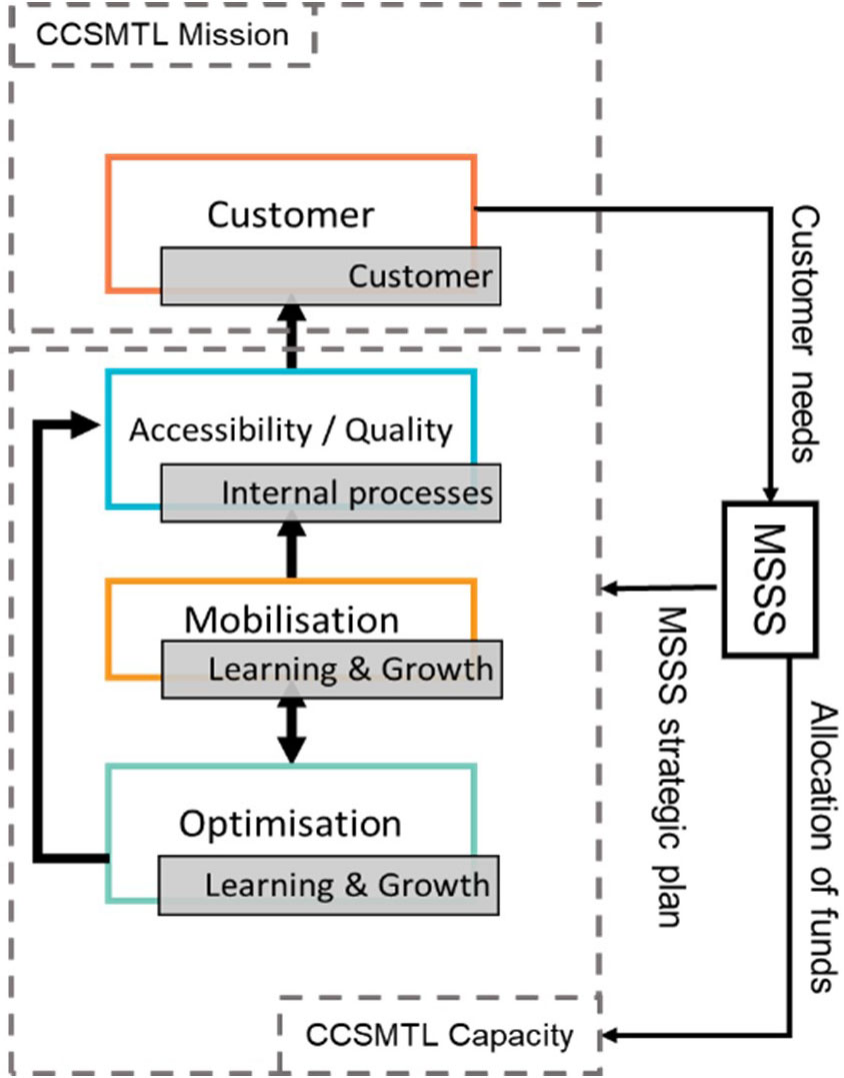
Proposed Balanced-QPM.

**Figure 6.**
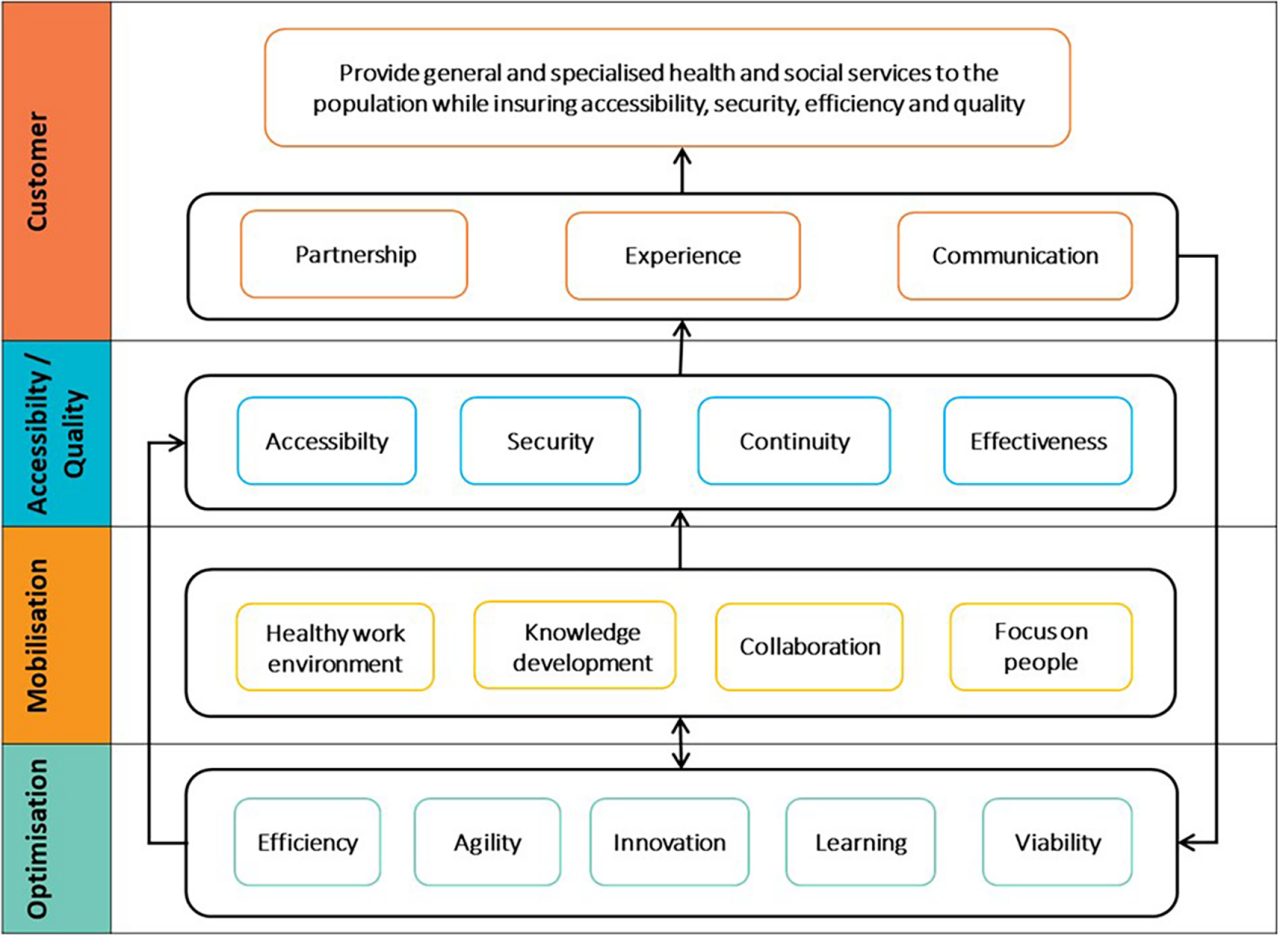
Generic strategy map proposed for the CCSMTL.

In this section, the results of phases II (SWOT analysis), III (Balanced-QPM and strategy map) and VI (indicator structure design and performance assessment) are presented. First, the results of the SWOT analysis are presented ( Table 4). After that, the Balanced-QPM ( Figure 5), the CCSMTL’s strategy map ( Figure 6), and the indicator structure system proposed for the CCSMTL are introduced. Finally, to illustrate how a strategy map could be built for a specific department, and how the performance indicator structure could be applied and the performance measured, the physical disability, intellectual disability, and autism spectrum disorder case, referred to as DI-TSA-DP, is used.

### Results of the SWOT analysis

From Table 4, we can see that one of the strengths identified is the well implementation of the QPM at the strategic level. There is strong interest by senior managers in aligning the organisation’s practices with QPM objectives. In addition, there are several practices put in place to ensure service quality and customer satisfaction and there is a real willingness to embed a customer-focused culture through the implementation of purposeful projects. The deployment and use (even partially at the moment of conducting this study) of the visual management system at different decision-making levels demonstrates a real desire to build an effective management and measurement performance system. We also identified that there are enough indicators in the accessibility/quality dimension. Another important aspect is that managers at different hierarchical levels have the possibility to propose performance indicators in compliance with general ministerial objectives (even though some indicators are imposed by the MSSS). Moreover, each department holds regular meetings, which helps to maintain communication within the departments and support the information cascade-escalation process.

While the QPM is well implemented at the strategic level, it is not sufficiently operationalised at the tactical and operational levels. We also identified that the QPM’s dimensions are unbalanced in terms of number of performance indicators used within each dimension. We find many indicators measuring the quality/accessibility dimension, but there is a lack of indicators in optimisation, mobilisation, and customer dimensions. There is also a lack of specific targets for those indicators. With the current performance management system, it is not possible for senior managers to track performance indicators at tactical/operational levels or to verify the overall performance without consulting a large amount of information. In addition, it is not possible to examine easily the performance of a specific service or department. There is a discontinuity in the assessment of performance between hierarchical levels. That is, each department/service measures its performance independently of the higher hierarchical level and other departments/services. We identified that dependencies and conflicts among some indicators exist, and this is challenging for managers to deal with. For example, there is a need to simultaneously reduce waiting time and improve direct service time (time with customers). An improvement in one indicator can decrease the other and vice versa. Regarding opportunities, the CCSMTL must continuously challenge itself to maintain/obtain accreditations and certifications such as PLANTREE, Accreditation Canada and to also comply with the MSSS’s strategic plans. Finally, like most organisations in a continuously changing socio-economic context, the CCSMTL might be threatened by pandemics such as COVID-19 or workforce shortage. These aspects can notably lead to disrupt service accessibility.

**Table 4.** Results of the SWOT analysis (Phase II).

	Strengths	Weaknesses
**Internal aspects**	- The QPM is well implemented at the strategic level. - Several practices ensuring high-quality services are implemented. - There is a willingness to embed a customer-focussed culture through the implementation of purposeful projects. - The visual management system is implemented at different decision-making levels. - There is a sufficient number of indicators within accessibility/quality dimension. - CCSMTL managers have a certain flexibility in proposing performance indicators for their departments. - There is good communication within the organisation.	- The QPM is known at the tactical and operational levels, but not used for performance management and measurement purposes. - The QPM dimensions are unbalanced in terms of number of indicators within each dimension. - There is a lack of global and aggregated indicators. - It is not possible for senior managers to easily examine the performance of a specific service or department. - There is a discontinuity in the assessment of performance between the hierarchical levels. - The information presented in dashboards is not well discussed. - Some indicators are interdependent, and other ones are conflictual.
**External aspects**	**Opportunities**	**Threats**
	- Several opportunities are provided by institutions such as PLANTREE, Agrément Canada, MSSS, etc. to implement best performance management practices.	- Socio-economic context (pandemics such as COVID-19 and population aging). - Workforce shortage issue.

### Balanced-QPM, strategy map and indicator structure system (CCSMTL level)

Figure 5 shows the dimensions of the proposed Balanced-QPM. BSC’s customer perspective corresponds to the customer dimension of the QPM. BSC’s internal process perspective is equivalent to the accessibility/quality dimension in the QPM. Learning & growth axis is found in both mobilisation and optimisation dimensions of the QPM. Learning & growth presents three main components: human capital, information capital, and organisational capital. Human capital refers to the management of skills and behaviours of employees. This aspect is part of the mobilisation dimension of the QPM (use of skills and talents of everyone in the organisation, partners, customers, and their families, encouraging personal development and commitment to accomplish the mission of the CCSMTL). The mobilisation dimension takes the third position in the Balanced-QPM. Organisational capital is defined as the organisation’s ability to align employee objectives with the strategy while information capital is the way organisations use their information systems, networks, and databases to support accomplishing their operations. Both aspects are part of the optimisation dimension of the QPM. We recall that optimisation dimension aims at continuous improvement and innovation to ensure, over time, that the services offered are adapted to the needs of customers. BSC’s financial perspective is also part of optimisation dimension. Kaplan & Norton (1999) stated that the financial perspective serves as a constraint, not an objective, for non-profit organisations such as healthcare systems. Therefore, optimisation dimension, takes the last position in the Balanced-QPM.

Additionally, Figure 5 shows global causal links between the four dimensions. The optimisation dimension has direct impact on the accessibility/quality dimension. There is also a mutual relationship between optimisation and mobilisation. Mobilisation has an impact on the accessibility/quality of services provided by the CCSMTL, which in turn has direct impact on customer satisfaction. Finally, customers put pressure on the MSSS to provide the necessary funding supporting the CCSMTL capacity in terms of optimisation, mobilisation, accessibility/quality aspects.

Figure 6 shows the strategy map of the CCSMTL. The causal links between the four dimensions shown in the figure correspond to those identified in Figure 5. For a customised strategy maps, e.g. DI-TSA-DP program, these causal links are defined more precisely (see Figure 8 for an example). The CCSMTL’s strategy map is the basis for building “customised” strategy maps for the different departments of the organisation.

**Figure 7.**
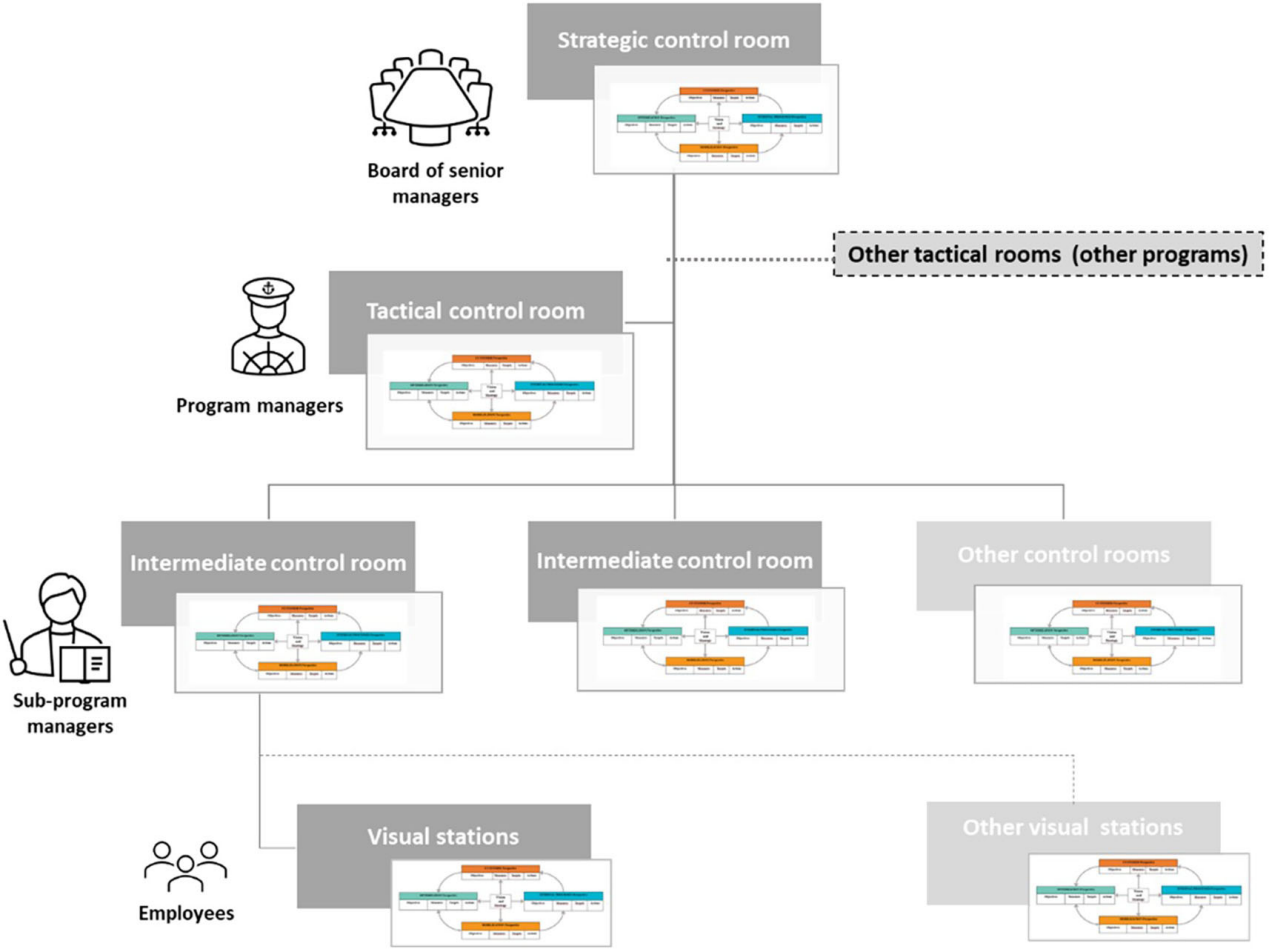
Proposed hierarchy and network-based scorecard structure. Balanced-QPM scoredcard at the visual station level.

Finally, Figure 7 shows the indicator structure system of the CCSMTL. We recall that this structure is based on the CCSMTL organisational chart and visual management system (CCSMTL’s network of control rooms). We propose to use a network of scorecards presenting the performance indexes and indicators of each dimension of the Balanced-QPM of the different departments of the CCSMTL to evaluate and discuss the performance at different hierarchical levels of the organisation. In other words, information on performance would be presented in scorecards (For an example, see Figure 10) and discussed in CCSMTL’s control rooms, and then would flow-up from operational to tactical and strategic levels (through tactical and strategic control rooms) or flow-down from strategic to tactical and operational levels, following the cascade-escalation mechanism used in the current visual management system of the CCSMTL.

### DI-TSA-DP case

DI-TSA-DP program offers services to 2,200 customers and receives approximately 10,000 service requests per year, mainly in the South-Centre of Montreal region and throughout the province of Quebec. 2,150 employees and 58 physicians work in this department. It includes three main sub-programs; DI-TSA, which provides services to customers with an intellectual disability or autism spectrum disorder, DP, which provides services to customers with a physical disability, and RMVS, which provides rehabilitation services in substitute living environments such as elderly homes. DI-TSA-DP mission is to provide specific, specialised and super-specialised habilitation and rehabilitation services to its customers to promote their integration and social participation.

At the moment of conducting this study, DI-TSA-DP has put in place one tactical room (top management level of the program), and three intermediate control rooms at the sub-program level (DI-TSA, DP, and RMVS). Operational rooms are established at the team unit level within the three sub-programs. Control room deployment at the team unit level was still in progress. Therefore, each sub-program had only reached a certain deployment rate, which is seen as a measure of performance-culture maturity level in the CCSMTL. Following the recommendation of our collaborators, we focussed on DP sub-program, since it had the highest deployment rate. The approach can be applied to the two other sub-programs as well. DP includes three services: AT (Technical Aids), which provides special assistance devices (prosthesis, wheelchair, etc.), LN (Locomotor-Neurology), which provides services to customers with physical disabilities due to neurological or locomotor disorders, and SL (Sensory-Language), which provides services to customers with linguistic or sensory disabilities. We focus on LN service, again based on its operational room deployment rate (100%). There are nine care team units within LN service, and each unit has its own operational room. We worked in close collaboration with two teams, Locomotor team (A-BOG), which provides services for customers with an amputation or serious orthopaedic injuries, and Neurological team (AVC), which provides services for customers who have had a stroke.

### DI-TSA-DP strategy map

**Figure 8.**
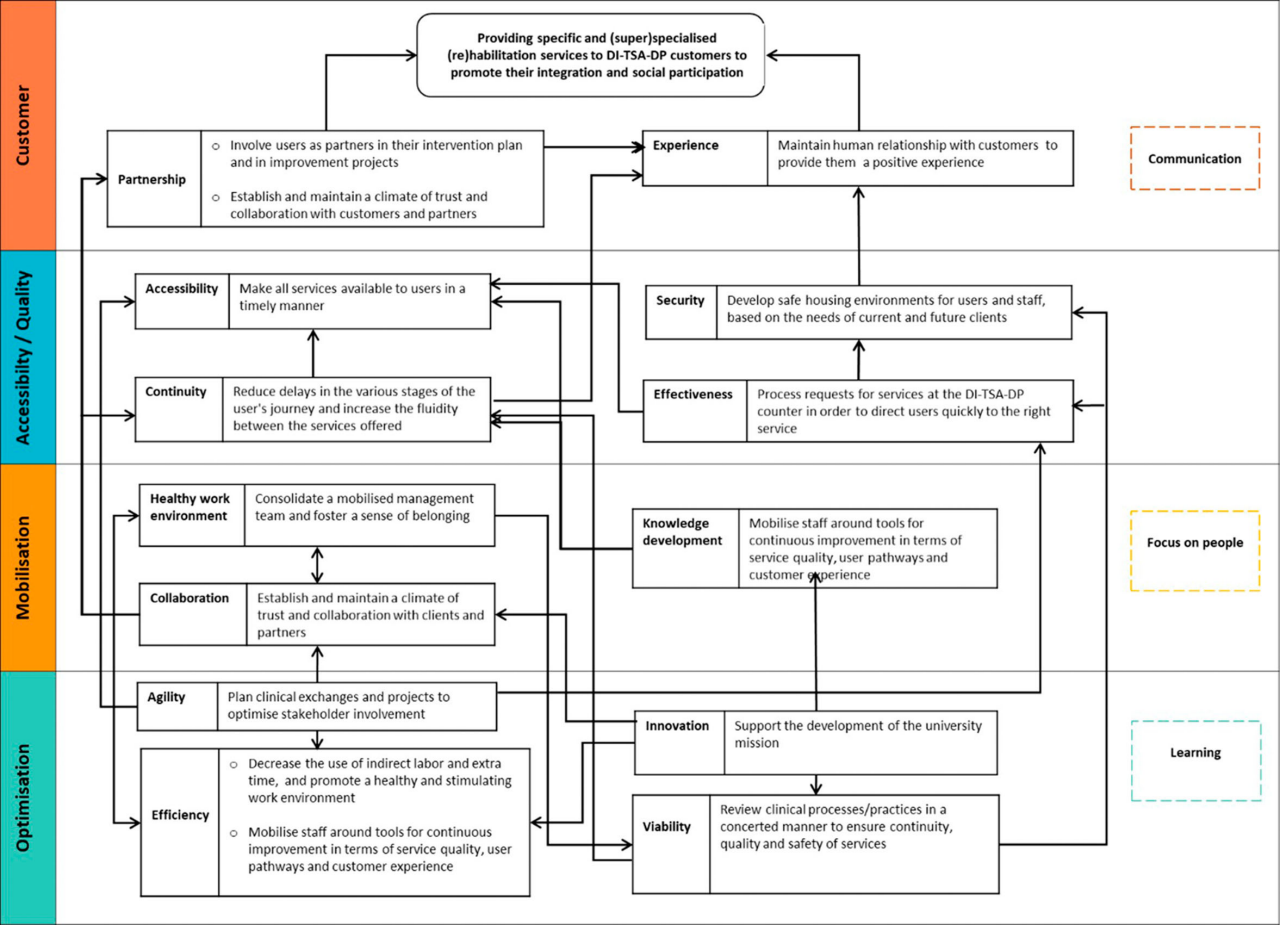
Proposed strategy map for DI-TSA-DP program.

The proposed strategy map for DI-TSA-DP ( Figure 8) is consistent with the generic strategy map ( Figure 6). It was co-created with the DI-TSA-DP program executive manager.

It illustrates objectives determined following the QPM sub-dimensions (including the primary objective) and causal links established between these objectives and/or sub-dimensions. The objectives selected are based on the internal DI-TSA-DP strategic vision 2018-2021 provided by DI-TSA-DP executive manager. Each objective was checked against the sub-dimensions of the QPM to identify to which one it is related. In total 16 objectives were classified within the sub-dimensions and dimensions of the QPM. The DI-TSA-SP mission was chosen as the primary objective and added in the customer dimension. Since no objective exists for customer experience, a new objective was proposed based on customers’ definition of this sub-dimension ( Table 1). Additional objectives should be set for this sub-dimension and other ones that currently do not present any specific objectives (i.e., communication, learning and focus on people).

Examples of causal links in the strategy map, are the relationship between objectives within customer sub-dimensions (‘partnership’ and experience’) and the primary objective (DI-TSA-DP mission). It is clear that positive customer experience, customer involvement as a partner in the system, and providing a climate of trust for the customers contribute to achieving DI-TSA-DP mission. It is interesting to notice that causal links also exist between sub-dimensions/objectives within the same dimension. As an example, partnership objectives contribute to achieving customer experience objectives. That is, when customers are involved as partners in the system, or when a climate of trust and collaborations is established with them, they are likely to have a positive experience. Some objectives/sub-dimensions mutually impact each-other, e.g. efficiency (optimisation dimension) and healthy work environment (mobilisation dimension). Reducing indirect labour and extra working hours (efficiency sub-dimension) leads to a healthy work environment, which in turn has a positive impact on efficiency.

The strategy map was key for aligning DI-TSA-DP vision and strategy with the four dimensions/sub-dimensions of the QPM. This step helped to bring clarity and logic to the strategic objective determination process in relation to the QPM dimensions/sub-dimensions and performance measurement. Based on the strategy map, it was also possible to align LN service performance indicators with the four dimensions of the QPM through the strategic objectives and sub-dimensions selected as shown in the next paragraphs.

## DI-TSA-DP performance measurement (phase IV)

### Indicator structure design and performance measurement

We conducted five working meetings of two hours each with different DI-TSA-DP managers. We started with the operational level (A-BOG and AVC) and moved to the tactical level (LN and DP service and sub-program, respectively), and finally, to the highest level (DI-TSA-DP program). The following paragraphs present the process of applying the developed PMMS to DI-TSA-DP case.

### Step 1: Indicator system structure

**Figure 9.**
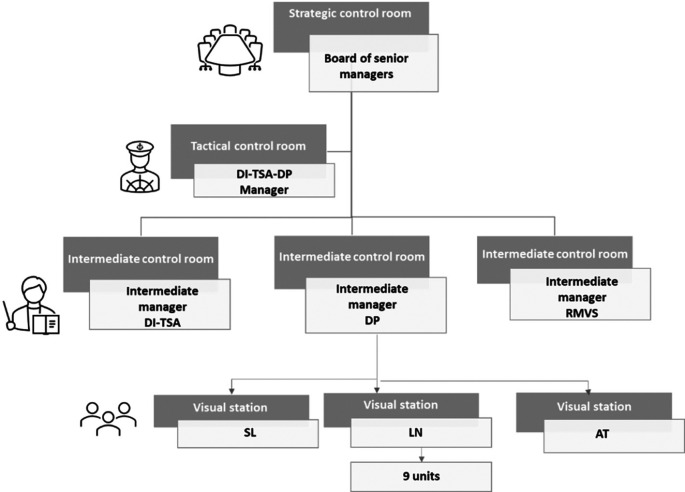
Simplified indicator system structure proposed for DI-TSA-DP.

Figure 9 shows the indicator system structure proposed for DI-TSA-DP. It is based on DI-TSA-DP organisational chart (and visual management system) and is part of the CCSMTL indicator system structure ( Figure 7). Figure 9 shows only the departments needed to illustrate our approach. We distinguish four levels: 1) CCSMTL strategic level, 2) tactical level, which includes DI-TSA-DP program and DI-TSA, DP, and RMVS sub-programs, and 3) operational level (within DP sub-program) that includes SL, LN, and AT teams.

**Figure 10.**
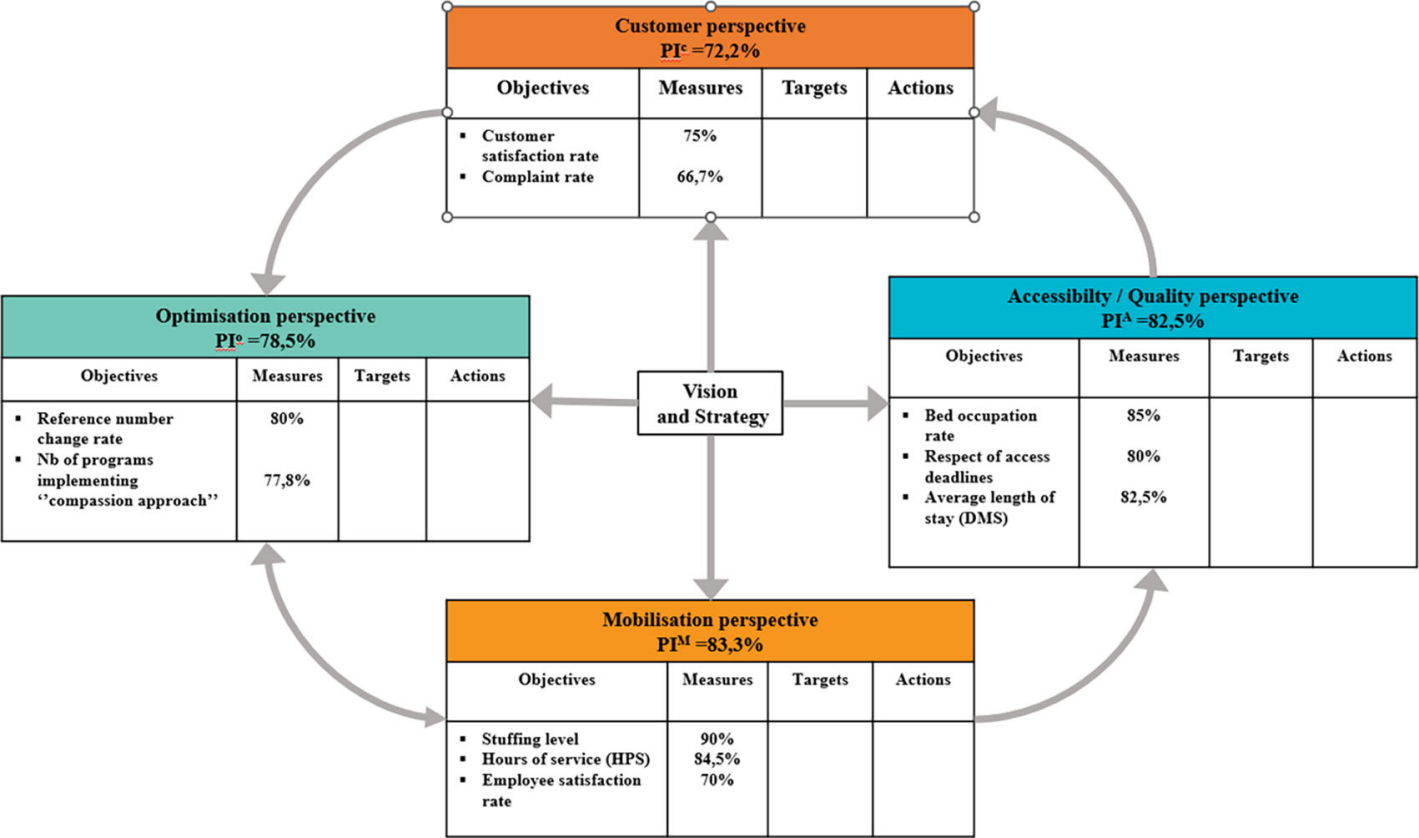
LN scorecard.

Figure 9 also shows the network of control rooms deployed in these different levels. The performance indicator structure follows the organisational chart and control room network structure. They are presented in the form of scorecards following the four Balanced-QPM dimensions (indicators, values and targets, performance indexes). For an example of a scorecard, see Figure 10.

### Step 2: Indicator identification and selection

To apply the rest of Phase IV steps, LN service was considered, and the process was performed in collaboration with LN coordinator. First, indicators being used by LN service were classified according to the four dimensions of the QPM and the strategy map’s sub-dimensions/objectives. Next, we checked the relevance of each performance indicator (against the criteria specificity, validity, simplicity, relevance, sustainability, reliability, and utility). When an indicator does not meet all criteria, it is either changed for another indicator that satisfies the criteria, or more information is gathered to ensure that the indicator meets all criteria. As an example, indicator ‘Customer satisfaction rate’ ( ${\mathrm{Xan}}_{1}^{C}$ ) ( Table 5), which was proposed to measure customer experience sub-dimension objective, did not meet simplicity criterion. LN managers did not have information on customers’ satisfaction and did not put in place specific methods to collect this information. To address this issue, we relied on the results of a survey used to collect the feedback of customers regarding their satisfaction level regarding experience and service quality. Table 5 shows the 10 final indicators selected for LN service.

**Table 5.** Indicators selected and their values after normalisation (LN service).

Dimensions	Measured sub-dimensions / Objectives	Indicator description	Indicator notation ( ${\mathrm{Xbn}}_{i}^{\dim}$ )	${\mathrm{Xbn}}_{i}^{\dim}$ value	Targets (Targeti)	Worst value (min _i_)	Normalized indicator ( $X_{i}^{\dim}$ ) value
**Customer**	Experience	Customer satisfaction rate	${\mathrm{Xbn}}_{1}^{C}$	75%	90%		75%
	Experience	Complaint rate	${\mathrm{Xbn}}_{2}^{C}$	60%	40%	100%	66.7%
**Accessibility/Quality**	Accessibility	Bed occupancy rate	${\mathrm{Xbn}}_{1}^{A}$	85%	95%		85%
	Accessibility Continuity Effectiveness	Respect of access deadlines	${\mathrm{Xbn}}_{2}^{A}$	80%	90%		80%
	Continuity	Average length of stay (DMS)	${\mathrm{Xbn}}_{3}^{A}$	17 days	10 days	50 days	82.5%
**Mobilisation**	Healthy work environment Collaboration	Staffing level	${\mathrm{Xbn}}_{1}^{M}$	90%	98%		92%
	Healthy work environment	Ratio of salary insurance hours	${\mathrm{Xbn}}_{2}^{M}$	7%	5,9%	13%	84.5%
	Healthy work environment Collaboration	Employee satisfaction rate	${\mathrm{Xbn}}_{3}^{M}$	70%	90%		70%
**Optimisation**	Innovation	Reference number change rate	${\mathrm{Xbn}}_{1}^{O}$	80%	90%		80%
	Innovation	Number of programs that have implemented the ‘compassion’ approach	${\mathrm{Xbn}}_{2}^{O}$	7 programs	9 programs	0 programs	77.8%

### Step 3: Indicator normalisation

The columns of Table 5 show the indicators measuring the objectives (and therefore sub-dimensions) selected in previous step, their notations, their values before normalisation, their targets, their worst values, and their values after normalisation (obtained by using Equation (2)).

Due to confidentiality reasons, all real values of the indicators have been modified. Note that indicators that are already expressed as percentages before normalisation and which are aimed to be maximised to reach the desired target have not been normalised. The worst value is set to zero for those indicators (e.g. Customer satisfaction rate, Bed occupancy rate, and Respect of access deadlines). Moreover, indicators before normalisation meant to be minimised to reach their target have been converted into indicators to be maximisation after normalisation (e.g. Complaint rate and Ratio of salary insurance hours).

### Steps 4 and 5: Indicator weighting and aggregation

Pairwise comparisons of normalised indicators within each QPM dimension were performed with the LN coordinator. The values obtained were entered in a VBA program that checks the consistency of the matrices and generates the weights following AHP algorithm (see Equations (4) to (6)). The final results that represent the weights of the indicators are presented in Table 6.

**Table 6.** Calculating performance indexes for LN sub-service.

Dimension	Customer		Accessibility/Quality			Mobilisation			Optimisation	
$X_{i}^{\dim}$	$X_{1}^{C}$	$X_{2}^{C}$	$X_{1}^{A}$	$X_{2}^{A}$	$X_{3}^{A}$	$X_{1}^{M}$	$X_{2}^{M}$	$X_{3}^{M}$	$X_{1}^{O}$	$X_{2}^{O}$
$X_{i}^{\dim}$ (%)	75%	66.7%	85%	80%	82.5%	92%	84.5%	70%	80%	77.8%
$W_{i}^{\dim}$	067	0.33	0.44	0.44	0.11	0.25	0.50	0.25	0.33	0.67
Performance index ( ${\mathrm{PI}}_{\mathrm{LN}}^{\dim}$ )	72.2%		82.5%			82.3%			78.5%	

Step 5 enables us to calculate aggregated performance indexes for each dimension that can be used by the service managers or by higher hierarchy level departments (DP sub-program and DI-TSA-DP programs in our case) to evaluate how the service performs globally in each dimension of the QPM. For instance, if the global performance of a given dimension is not satisfying, the performance indicators within that dimension should be analysed in more detail to identify the problem and take actions to improve the situation. The results of calculating the four performance indexes $\left({\mathrm{PI}}_{\mathrm{LN}}^{C},{\mathrm{PI}}_{\mathrm{LN}}^{A},{\mathrm{PI}}_{\mathrm{LN}}^{M},{\mathrm{PI}}_{\mathrm{LN}}^{O}\right)$ of LN are given in Table 6. Each performance index corresponds to one dimension of the Balanced-QPM. Again, the VBA program is used to automate these calculations following AHP algorithm. These aggregated performance indexes are finally visualised on scorecard so that managers can examine each performance dimension and put actions to improve the critical ones. Figure 10 illustrates the LN scorecard with the performance indexes selected for LN for each QPM dimension and performance indicators for each dimension.

It is worth noting that, when using AHP, we identified a compensation effect between indicators that results from aggregating two (or more) indicators having opposite values, which is not desired. For instance, within Mobilisation dimension, the normalised indicator ‘Staffing level’ ( $X_{1}^{M}$ ) has a value of 92% while the normalised indicator ‘Employee satisfaction rates ( $X_{13}^{M}$ ) has a value of 70%. Their aggregation together with the normalised indicator ‘Ratio of salary insurance hours’ ( $X_{2}^{M}$ ) (value of 84.5%), leads to a performance index with a value of 82,3% ( ${\mathrm{PI}}_{\mathrm{LN}}^{M}$ ) ( Table 6). It is easy to see that the high value of ‘Staffing level indicator’ compensates the lower value of ‘Employee satisfaction rate’ indicator. This result also depends on the weights assigned to the three indicators (0.25, 0.25, and 0.50, respectively in our case).

## Discussion

In this study, we have proposed a comprehensive and quantitative approach to develop or improve a PMMS. We have illustrated our approach by using CCSMTL’s current performance management model, the QPM. Recent studies in the literature recognise the importance and at the same time the complexity of measuring performance in healthcare organisations. However, most of these studies are descriptive and do not propose PMMSs capable of effectively structuring and measuring performance. The development of a strategy map enables the operationalisation of an organisation’s staregy and identifying relevant performance indicators aligned with the strategy. In other words, the strategy map plays a pivotal role in translating the organisation’s vision and strategy into performance objectives and measurable indicators. Moreover, in the literature, the BSC and AHP method (and its variants, e.g. ANP) have been used to identify global indicators, ranking and prioritizing those indicators, or compare performance across different healthcare organisations. Our approach allows managers to measure performance at different hierarchical levels of an organisation and aggregate the information to facilitate global performance assessment and support decision-making at the strategic level. This is an important problem faced by many healthcare organisations, notably by large institutions, yet it not well addressed in the literature. In the CCSMTL context, aggregating the performance indicators and calculating one performance index for each dimension by using AHP was well appreciated by the managers. In addition, our approach contributed to balancing the four dimensions of the CCSMTL’s QPM by ensuring that a sufficient number of performance indicators measure each dimension. Finally, performance indicators selection relied on a transparent and rigorous process that involved the participation of managers. This contributes to foster positive changes in the organisation regarding perfrormance measurement and performance culture in general.

CCSMTL case study helped us to better understand the challenges and difficulties managers may face during strategy map construction and PMMS development/implementation processes. Regarding the strategy map, iterative meetings with managers were necessary to build the version presented in this work (DI-TSA-DP program). More collaboration is required to improve this preliminary version (i.e. determining more precisely the strategic objectives and causal links between them). We have also observed that choosing targets for the indicators was not an easy task for managers. In fact, most existing indicators are associated with organisational targets that are set by strategic management. Due to this, it is challenging for managers to select reasonable targets for new indicators.

In addition, deep discussions took place between managers during the processes of comparing indicators to each other to agree on common preferences. This shows the difficulty of making common pair-wise comparisons and deriving the right weights, and therefore the right performance index values. This aspect was also observed in Boukherroub *et al.* (2017). It deserves to be addressed in future work, for instance, by considering group-decision-making techniques in the weighting process or by discussing this issue at the strategic management to set clear guidelines to support managers at different hierarchical levels in defining the weights that best reflect the vision of the organisation. Dependencies among aggregated indicators might also occur. As an example, in psychosocial services, waiting time and customer service time evolve in opposite directions as an improvement in one indicator decreases the other and vice versa (i.e. when a social worker spends more time with his/her current customers, new customers assigned to him/her will wait longer before they can be met) ( Boukherroub *et al.*, 2022). Therefore, methods that address this issue are also required. Typically, the ANP technique, which is a generalisation of AHP, is appropriate for this situation. BWM (Best Worst Method) ( Singh & Singh, 2020) and MACBETH (Measuring Attractiveness by a Categorical Based Evaluation Technique) ( Bana e Costa & Vansnick, 1994) are other possibilities.

This study has some limitations. Regarding data collection, more managers and employees coming from different services and hierarchical levels should have been included in the interview process. Due to the COVID-19 pandemic, it was not possible to include more people. Strategy maps should be built for all programs of the CCSMTL prior to indicator selection, validation, information aggregation, etc. to align the measured performance with strategic objectives. Brainstorming workshops should be carried out with managers and coordinators to establish more precisely their objectives and causal links between these objectives. More work is also required to design additional indicators. In particular, means of measuring and monitoring indicators over time need to be developed. Since AHP does not take into consideration potential interdependences between indicators, ANP, BWM, and MACBETH techniques could be explored. Undesired compensation effects might also occur in the aggregation process. Finally, it is not recommended to limit the use of performance indexes alone for monitoring performance. Operational indicators related for instance to emergency services are very important to monitor at the highest management level. In sum, more research is needed to refine our proposed approach.

## Conclusions and research perspectives

The CCSMTL has developed a multi-dimensional performance model (Quality-Performance Model - QPM), however, its implementation across the organisation is very challenging. This study proposes an approach that contributes to improving this model and its effective deployment. First, our proposed strategy map supports managers in identifying indicators that are aligned with their strategic objectives and vision. Second, the proposed PMMS helps them focus on all four dimensions of the QPM by explicitly linking performance indicators to each dimension. This, notably, supports proposing performance indicators within the customer dimension, which contributes to promoting customer-focussed culture. This would also allow departments to concretely measure their performance and compare themselves in a way that fosters competitiveness and anchors performance-oriented practices in the organisation. Third, the aggregated performance measures (performance indexes) that we propose to calculate based on AHP method will help managers at higher hierarchy levels to have a better visibility and understanding of the performance of operational teams, and make better decisions to support their improvement. Finally, our approach would support standardising performance management practices within the organisation. Our work contributes to the theory and practice by proposing a performance management and measurement approach adapted to large healthcare organisations, which is has been reported in practice and in the literature as a very challenging problem ( Moisan, 2019).

The following is a testimonial from the deputy director of organisational performance at the CCSMTL: *“As part of this project, several working sessions were held with stakeholders [CCSMTL managers]. These structured interviews enabled the research team to well capture the QPM and then develop their approach accordingly. The data [performance indicators] organisation structure is very relevant in the sense that it addresses both the dimensions and the sub-dimensions of the QPM, and it is declined according to the hierarchical administrative structure of the CCSMTL, thus matching with our approach of continuous improvement, which aims to dynamise the information cascade-escalation process through all layers of the organisation. Finally, the rigorous process of validating and weighting the indicators was carried out with the stakeholders, thus ensuring better reliability of the indicators developed, which will optimise their use to eventually support decision-making.”*

## Data Availability

No data are associated with this article.
