# Supplementary material for: Towards an Integrated Performance Management and Measurement System for healthcare organisations: a case study in Montreal
Source: F1000Res. 2024 Dec 9;12:1420. Originally published 2023 Oct 30. [Version 2] doi: 10.12688/f1000research.138430.2 (PMC11406139; doi:10.12688/f1000research.138430.2)
Supplement: Peer review history

# Reviewer report: Reviewer response for version 2

**DOI:** 10.5256/f1000research.169268.r347038

**Published:** 2024-12-23

**Authors:** Jean-Baptiste Gartner

**Affiliations:** 1 Universite Laval, Québec City, Québec, Canada

This study aims to develop a performance management and measurement system for a large healthcare organisation (integrated (University) centres of healthcare and social services) in Montreal, Quebec, Canada.

 Overall, the revisions have greatly improved the document. However, there are still some points that need to be improved before it can be accepted.

*INTRODUCTI* *ON/LITERATURE REVIEW*

*- The background and the context are clearly stated.*

*- *"Even though recent studies in the literature discuss performance management systems in healthcare organisations and recognise both the importance and the complexity of measuring performance, most of them are descriptive and do not propose PMMSs capable of effectively structuring and measuring the performance of healthcare organisations. *" *This statement must be supported by at least one reference. Otherwise, it appears to be a personal opinion.

*- *"The QPM (see Figure 1) is based on the MSSS strategic plan (2019-2023 period)" This statement does not work (the legal deposit of the document of the MSSS dates from after the article was published), remove or explain.

*LITTERATURE REVIEW*

 This part has been suitably enhanced.

*METHODS*

 The addition of the table is appropriate.

 The addition of details of the method is welcome.

*RESULTS*

 Given the number of data sources (interview, observation, focus group), it is surprising to have been left with a single case study description. With this in mind, it is unfortunate that assertions are not supported by your empirical material, for example :

 - "From Table 4, we can see that one of the strengths identified is the well implementation of the QPM at the strategic level". (This is an assertion, but the reader has no data to corroborate the assertion (interview excerpts, focus groups, changes in a quantitative/qualitative indicator, etc.). If the intention is to be descriptive only, this can remain unchanged.

*DISCUSSION*

 The discussion still does not contextualise the results with existing knowledge (present in your Literature review paragraph). Contribution of your article ?

*- Future work: Ok, your opening is relevant.*

Some typos and errors:

*Introduction:*

*- "*In the province of Qu **é** bec *" (Quebec)*

*- "*which fall under the responsibility of the Ministry of Healthcare and Social Services (Mi **m** istère de la Santé et des Services Sociaux".

Is the case presented with sufficient detail to be useful for teaching or other practitioners?

Partly

Is the work clearly and accurately presented and does it cite the current literature?

Partly

If applicable, is the statistical analysis and its interpretation appropriate?

Not applicable

Are all the source data underlying the results available to ensure full reproducibility?

No

Are the conclusions drawn adequately supported by the results?

Yes

Is the background of the case’s history and progression described in sufficient detail?

Yes

Reviewer Expertise:

Healthcare management research

I confirm that I have read this submission and believe that I have an appropriate level of expertise to confirm that it is of an acceptable scientific standard, however I have significant reservations, as outlined above.

# Reviewer report: Reviewer response for version 1

**DOI:** 10.5256/f1000research.151623.r219887

**Published:** 2024-09-16

**Authors:** Pascal Forget

**Affiliations:** 1 Université du Québec à Trois-Rivières, Trois-Rivières, Quebec, Canada

This article proposes an approach to facilitate the implementation of a performance management model in a government healthcare system.

 This article is really interesting and offer a pertinent contribution. It is clear and well written. The introduction, the literature, the method and discussion are clear and well presented.

 There is no specific point to address and the paper could be index as it is.

Is the case presented with sufficient detail to be useful for teaching or other practitioners?

Yes

Is the work clearly and accurately presented and does it cite the current literature?

Yes

If applicable, is the statistical analysis and its interpretation appropriate?

Not applicable

Are all the source data underlying the results available to ensure full reproducibility?

Yes

Are the conclusions drawn adequately supported by the results?

Yes

Is the background of the case’s history and progression described in sufficient detail?

Yes

Reviewer Expertise:

Industrial engineering, operation research, simulation

I confirm that I have read this submission and believe that I have an appropriate level of expertise to confirm that it is of an acceptable scientific standard.

# Reviewer report: Reviewer response for version 1

**DOI:** 10.5256/f1000research.151623.r238355

**Published:** 2024-04-04

**Authors:** Paulino Silva

**Affiliations:** 1 ISCAP Polytechnic of Porto, Porto, Porto District, Portugal

The article presents a very relevant topic with great added value, both for the organizational performance management literature and for management practitioners. The contribution of the article can be relevant for the body of knowledge. However, there are some methodological weaknesses that should be considered.

 The article claims to develop an integrated performance management system for healthcare organizations, but perhaps it would be more appropriate to define it as an improvement to the existing system. This is because it makes a diagnosis of the existing system and, through SWOT analysis, presents the main strengths, weaknesses, opportunities, and threats.

 The used case study seems too specific (Physical disability, intellectual disability, and autism spectrum disorder" of CIUSSS du Centre-Sud-de-l'île-de-Montreal, in the province of Québec, Canada) to be considered a reference of a performance management system in healthcare organizations.

 As the study began in 2019, and the interviews between May 2019 and September 2021, could the results have been too affected by the Covid-19 pandemic?

 The number of interviews seems to be very low (only 12) and the variety of jobs very limited (only managers).

 Consider including additional literature specifically related to performance management in healthcare organizations, such as : [1]

 Some “typos” in the article:

- The first paragraph of the methods section is incorrect, it should end with phases III and IV.
- The abbreviation for Ministry of Healthcare and Social Services presented in the article is MSSS and not MHSS, why?
- Customer-focused instead of customer-focussed in line 6 of the section Conclusions and research perspectives.

Is the case presented with sufficient detail to be useful for teaching or other practitioners?

Yes

Is the work clearly and accurately presented and does it cite the current literature?

Partly

If applicable, is the statistical analysis and its interpretation appropriate?

I cannot comment. A qualified statistician is required.

Are all the source data underlying the results available to ensure full reproducibility?

Partly

Are the conclusions drawn adequately supported by the results?

Yes

Is the background of the case’s history and progression described in sufficient detail?

Yes

Reviewer Expertise:

Management Accounting

I confirm that I have read this submission and believe that I have an appropriate level of expertise to confirm that it is of an acceptable scientific standard, however I have significant reservations, as outlined above.

## References

[1] : Performance management in primary healthcare services: evidence from a field study. *Qualitative Research in Accounting & Management* .2010;7(4) : 10.1108/11766091011094527 424-449 10.1108/11766091011094527

# Response

**Published:** 2024-10-28

**Authors:** NEILA EL ASLI

**Affiliations:** Industrial Engineering, University of Québec in Trois-Rivières, Trois-Rivières, Québec, Canada

**Reviewer #3: **APPROVED WITH RESERVATIONS

*The article presents a very relevant topic with great added value, both for the organizational performance management literature and for management practitioners. The contribution of the article can be relevant for the body of knowledge. However, there are some methodological weaknesses that should be considered.*

*The article claims to develop an integrated performance management system for healthcare organizations, but perhaps it would be more appropriate to define it as an improvement to the existing system. This is because it makes a diagnosis of the existing system and, through SWOT analysis, presents the main strengths, weaknesses, opportunities, and threats.*

**You are right. The PMMS (Performance Management and Measurement System) we proposed is an improved Quality-Performance Model (QPM) in the context of the CCSMTL. However, in the absence of a performance model, our approach (or at least part of it if we remove the SWOT analysis), could be used to develop a PMMS. We clarified this in the text in the new version of the manuscript at different levels:**

- ** We changed the title from “Developing an integrated ….” to “ Towrads an integarted …”.**
- **In the abstract, the first sentence is now: “This study proposes an approach for developing or improving performance management and measurement systems (PMMSs) for healthcare organisations.”.**
- **In the Introduction (last paragraph):  “…it proposes a four-phases based approach to support CCSMTL managers to improve and transform the QPM into a more balanced and effective performance management and measurement system (PMMS). … It can be used by theoreticians and practitioners as roadmap to develop or improve healthcare organisations’ PMMSs.**
- **In the Discussion section: ““In this study, we have proposed a comprehensive and quantitative approach to develop or improve a PMMS.”**
- **In the Conclusion section: “The CCSMTL has developed a multi-dimensional performance model (Quality-Performance Model - QPM), however, its implementation across the organisation is very challenging. This study proposes an approach that contributes to improving this model and its effective deployment.”**

*The used case study seems too specific (Physical disability, intellectual disability, and autism spectrum disorder" of CIUSSS du Centre-Sud-de-l'île-de-Montreal, in the province of Québec, Canada) to be considered a reference of a performance management system in healthcare organizations.*

**There are two levels of application of our PMMS. We applied Phases I, II, III, and partially IV to the entire CIUSSS, and then Phases III and IV to the program *Physical disability, intellectual disability, and autism spectrum disorder*. We have made changes in the new version of the manuscript to clarify this (please see the Results section). We agree that more work is needed before we can consider this system as a reference in healthcare organizations. We have acknowledged this limitation in the discussion Section, and as mentioned before, we also have changed the title.**  

*As the study began in 2019, and the interviews between May 2019 and September 2021, could the results have been too affected by the Covid-19 pandemic?*

**This is right, part of the data collection took place during the COVID-19 pandemic (2020). During this period, the process slowed down; it was difficult to reach people. This resulted in a lesser number of interviews than what we targeted initially. However, it is difficult to estimate the impact on the results though.**

 The number of interviews seems to be very low (only 12) and the variety of jobs very limited (only managers).

**As mentioned above, initially we targeted more interviews. Even though all the participants involved are managers, there is a certain variety considering that they don’t have the same function, hierarchy level in the organization, and they don’t come from the same programs/services (please see Table 3 in the new version of the manuscript). Employees were also met during the data collection phase. However, their knowledge level regarding performance measurement, notably in relation to the QPM, was too low or inexistant (in some services, there are no performance indicators put in place at all). This lack of knowledge at the operational level has been mentioned as a weakness in the SWOT analysis “The QPM is known at the tactical and operational levels, but not used for performance management and measurement purposes”. We appreciate your comment. We have discussed this as a limitation in the new version of the manuscript.**    

*Consider including additional literature specifically related to performance management in healthcare organizations, such as : [1]*

*1. Silva P, Ferreira A: Performance management in primary healthcare services: evidence from a field study. Qualitative Research in Accounting & Management. 2010; 7 (4): 424-449 Publisher Full Text*

**Thanks for this suggestion. We have included it in our literature review. We have also added three other references suggested by Reviewer #1.**

*Some “typos” in the article:*

*The first paragraph of the methods section is incorrect, it should end with phases III and IV.*

 Y **ou are right. It has been corrected.**

*The abbreviation for Ministry of Healthcare and Social Services presented in the article is MSSS and not MHSS, why?*

**MSSS stands for *Ministère de la Santé et des Services Sociaux* in French (official title of the Ministry). We will have added this information in the new version of the manuscript to avoid confusion.**

*Customer-focused instead of customer-focussed in line 6 of the section Conclusions and research perspectives.*

**Thanks, we have corrected it.**

# Reviewer report: Reviewer response for version 1

**DOI:** 10.5256/f1000research.151623.r253136

**Published:** 2024-03-28

**Authors:** Wolfgang Munar

**Affiliations:** 1 The George Washington University, Washington, District of Columbia, USA

This solid paper contributes to ongoing debates on designing performance measurement and management systems in healthcare organizations. The authors describe the process followed to complete the system's design (i.e., situational analysis, SWOT analysis, and literature review), leading to the development of a performance indicator framework. An indicative application of the resulting design was then applied to the CIUSSS program.

 The main two areas to consider for improving the paper are described below.

 1. While the authors recognize the complexity inherent to PMM system design (and implementation), there is insufficient analysis/discussion of the proposed design's strengths and limitations to address such complexity (e.g., interdependence and recurrence between indicators, gaming, users' cognitive biases, etc.).  

 2. The authors' use of the concept of "implementation" requires clarification. While the paper clearly presents a prototype for a PMM system, it cannot be said that it describes its implementation.

Is the case presented with sufficient detail to be useful for teaching or other practitioners?

Yes

Is the work clearly and accurately presented and does it cite the current literature?

Yes

If applicable, is the statistical analysis and its interpretation appropriate?

Not applicable

Are all the source data underlying the results available to ensure full reproducibility?

Yes

Are the conclusions drawn adequately supported by the results?

Partly

Is the background of the case’s history and progression described in sufficient detail?

Yes

Reviewer Expertise:

Health policy and systems research, and theory-driven evaluation.

I confirm that I have read this submission and believe that I have an appropriate level of expertise to confirm that it is of an acceptable scientific standard, however I have significant reservations, as outlined above.

# Response

**Published:** 2024-10-28

**Authors:** NEILA EL ASLI

**Affiliations:** Industrial Engineering, University of Québec in Trois-Rivières, Trois-Rivières, Québec, Canada

This solid paper contributes to ongoing debates on designing performance measurement and management systems in healthcare organizations. The authors describe the process followed to complete the system's design (i.e., situational analysis, SWOT analysis, and literature review), leading to the development of a performance indicator framework. An indicative application of the resulting design was then applied to the CIUSSS program.

 The main two areas to consider for improving the paper are described below.

 1. While the authors recognize the complexity inherent to PMM system design (and implementation), there is insufficient analysis/discussion of the proposed design's strengths and limitations to address such complexity (e.g., interdependence and recurrence between indicators, gaming, users' cognitive biases, etc.).  

 Author Response: Thanks for this comment. We have improved the discussion section in the new version of the manuscript.

 2. The authors' use of the concept of "implementation" requires clarification. While the paper clearly presents a prototype for a PMM system, it cannot be said that it describes its implementation.

 Author Response: We agree that the us of the term “implementation” in the text can be confusing.  We clarified this in the new version of the manuscript at different levels:

- We changed the title from “Developing an integrated ….” to “ Towrads an integarted …”.
- In the abstract, the first sentence is now: “This study proposes an approach for developing or improving performance management and measurement systems (PMMSs) for healthcare organisations.”.
- In the Introduction (last paragraph):  “…it proposes a four-phases based approach to support CCSMTL managers to improve and transform the QPM into a more balanced and effective performance management and measurement system (PMMS). … It can be used by theoreticians and practitioners as roadmap to develop or improve healthcare organisations’ PMMSs.
- In the Discussion section: ““In this study, we have proposed a comprehensive and quantitative approach to develop or improve a PMMS.”
- In the Conclusion section: “The CCSMTL has developed a multi-dimensional performance model (Quality-Performance Model - QPM), however, its implementation across the organisation is very challenging. This study proposes an approach that contributes to improving this model and its effective deployment.”

# Reviewer report: Reviewer response for version 1

**DOI:** 10.5256/f1000research.151623.r253135

**Published:** 2024-03-09

**Authors:** Jean-Baptiste Gartner

**Affiliations:** 1 Universite Laval, Québec City, Québec, Canada

**Comments to the author(s)**

 This study aims to develop a performance management and measurement system for a large healthcare organisation (integrated (University) centres of healthcare and social services) in Quebec, Canada.

INTRODUCTION/LITERATURE REVIEW

 - The background and the context are clearly stated.

 - However, the construction of your introduction between PMMS literature and context needs to be better realized to better accompany the reader. Indeed, the construction of your introduction goes from:

 1. Contextualizing your case

 2. Recent cases in literature

 3. Model of QPM and its deployment

 4. Literature review on PMS.

 I invite you to review the structure of the introduction. It might be relevant to merge the literature review section with the cases described in the literature (2 and 4), to conclude your introduction with the QPM model and its implementation issues, justifying your study. And then, conclude with the aim of the study.

 Apart from the structure, certain points could be enhanced:

 - Avoid talking about the methods and results of the study in your introduction (paragraph “This study contributes to addressing”).

 - To further strengthen your literature:

 PMMS:

 Ferreira A, et. al. 2009 (Ref 1)

 Solomon R, et.al. 2013 (Ref 2)

 Québec context:

 Fache P, et. al. 2016 (Ref 3)

METHODS

 The methodology is interesting and diverse. Some points could be improved or better justified:

 - Data collection (Phase I): The material should be better described and quantified (approximate total duration of observations, socio-demographic characteristics of interviewees). A table summarizing the material collected would be a great help. In addition, you do not specify the methodology for collecting information (notebook or reflective notebook for the observations, Semi-structured interviews (recorded or not)). Finally, the interview guide should be included as an appendix.

 - Data analysis (Phase II): Using SWOT to structure your results is very interesting. But you say nothing about the method of analysis and synthesis (thematic coding by two independent authors and comparison of results? or a coder and then pooling). Here you need to focus on the method description. Have you used methods to ensure the robustness of the analysis (internal and external validity, data triangulation, consistency)? If so, please specify. Table 3 and the following paragraph should be put in the results section, as it is results.

 - Balanced-QPM development (Phase III) and Indicator structure design and performance measurement (Phase IV): Stay with the process (the method) and keep the results for the results section.

RESULTS

 Your results are interesting and relevant. Create a results section separate from the methods section.

DISCUSSION

 The discussion needs to be further enriched and your results better compared with current knowledge (recent cases in literature and literature review).

 - From my point of view, the first two paragraphs of the discussion are still part of the results (implementation results).

 - Future work: Ok, your opening is relevant.

Some typos and errors:

 Introduction: “The Coordinating Committee, which is composed of a limited number of managers, aims at insuring” (ensuring?)

 Methods: “Due of this, it is challenging” (to)

 Conclusion: “which is has been reported in practice and in the literature as a very challenging problem”

In summary:

 - The topic is interesting, and the study mobilizes a wealth of material to propose results relevant to the field.

 - There are several study structuring issues that need to be addressed (structure of introduction, absence of results title, structure of discussion. A number of tips are given for this purpose.

 - A number of methodological points need to be reinforced in order to fully convince the reader.

 - The literature could be strengthened to develop the discussion on the contribution of this study. Proposals to this effect have also been made.

 Good luck for the next steps.

Is the case presented with sufficient detail to be useful for teaching or other practitioners?

Partly

Is the work clearly and accurately presented and does it cite the current literature?

Partly

If applicable, is the statistical analysis and its interpretation appropriate?

Not applicable

Are all the source data underlying the results available to ensure full reproducibility?

No

Are the conclusions drawn adequately supported by the results?

Yes

Is the background of the case’s history and progression described in sufficient detail?

Yes

Reviewer Expertise:

Healthcare management sciences, Performance management systems, Care pathways optimization, Learning Health Systems

I confirm that I have read this submission and believe that I have an appropriate level of expertise to confirm that it is of an acceptable scientific standard, however I have significant reservations, as outlined above.

## References

[1] : The design and use of performance management systems: An extended framework for analysis. *Management Accounting Research* .2009;20(4) : 10.1016/j.mar.2009.07.003 263-282 10.1016/j.mar.2009.07.003

[2] : Measuring quality at a system level: an impossible task? The Toronto Central LHIN Experience. *Healthc Q* .2013;16(4) : 10.12927/hcq.2014.23654 36-42 10.12927/hcq.2014.23654 24485242

[3] : The Search is on for Coherent Performance Measurement in Healthcare Organizations. Has Quebec Reached a Crossroads?. *Healthc Policy* .2016;11(4) :60-9 27232237 PMC4872553

# Response

**Published:** 2024-10-18

**Authors:** NEILA EL ASLI

**Affiliations:** Industrial Engineering, University of Québec in Trois-Rivières, Trois-Rivières, Québec, Canada

In the following, you will find our responses in **bold style.**

*This study aims to develop a performance management and measurement system for a large healthcare organisation (integrated (University) centres of healthcare and social services) in Quebec, Canada.*

*INTRODUCTION/LITERATURE REVIEW*

*- The background and the context are clearly stated.*

*- However, the construction of your introduction between PMMS literature and context needs to be better realized to better accompany the reader. Indeed, the construction of your introduction goes from:*

*1. Contextualizing your case*

*2. Recent cases in literature*

*3. Model of QPM and its deployment*

*4. Literature review on PMS.*

*I invite you to review the structure of the introduction. It might be relevant to merge the literature review section with the cases described in the literature (2 and 4), to conclude your introduction with the QPM model and its implementation issues, justifying your study. And then, conclude with the aim of the study.*

**Thanks for this suggestion. Parts 3 and 4 are normally two distinct sections separated from the Introduction. The formatting of the journal merged them with the Introduction. We have corrected this. We agree that it is better to merge parts 2 and 4 and to conclude the introduction with the QPM model and the aim of the study. Please see the changes we have made in the new version of the manuscript.**

*Apart from the structure, certain points could be enhanced:*

*- Avoid talking about the methods and results of the study in your introduction (paragraph “This study contributes to addressing”).*

**Thanks for this suggestion. We have removed the details concerning the approach proposed as well as the description of the results in the new version of the manuscript.**

*- To further strengthen your literature:*

*PMMS:*

*Ferreira A, et. al. 2009 (Ref 1)*

*Solomon R, et.al. 2013 (Ref 2)*

*Québec context:*

*Fache P, et. al. 2016 (Ref 3)*

*1. Ferreira A, Otley D: The design and use of performance management systems: An extended framework for analysis. Management Accounting Research. 2009; **20** (4): 263-282* *Publisher Full Text*

*2. Solomon R, Damba C, Bryant S: Measuring quality at a system level: an impossible task? The Toronto Central LHIN Experience.Healthc Q. 2013; **16** (4): 36-42* *PubMed Abstract* *|* *Publisher Full Text*

*3. Fache P, Sicotte C, Minvielle É: The Search is on for Coherent Performance Measurement in Healthcare Organizations. Has Quebec Reached a Crossroads?. Healthc Policy. 2016; **11** (4): 60-9* *PubMed Abstract*

**Thanks for these references. We have added them in the literature review section of the new version of the Manuscript.**

*METHODS*

*The methodology is interesting and diverse. Some points could be improved or better justified:*

*- Data collection (Phase I): The material should be better described and quantified (approximate total duration of observations* *, socio-demographic characteristics of interviewees). A table summarizing the material collected would be a great help.*

*In addition, you do not specify the methodology for collecting information (notebook or reflective notebook for the observations, Semi-structured interviews (recorded or not)). Finally, the interview guide should be included as an appendix.*

**In the new version of the manuscript, we have added a table summarizing the data collection methods used for conducting phase I and short descriptions (Table 3). We also referred the reader to a work presenting more in detail on the collection data methods in the text (Ben Fradj, 2020).**

**Unfortunately, the journal does not allow including any appendix.**

*- Data analysis (Phase II): Using SWOT to structure your results is very interesting. But you say nothing about the method of analysis and synthesis (thematic coding by two independent authors and comparison of results? or a coder and then pooling). Here you need to focus on the method description. Have you used methods to ensure the robustness of the analysis (internal and external validity, data triangulation, consistency)? If so, please specify. Table 3 and the following paragraph should be put in the results section, as it is results.*

**We have added more information in the text to describe how we conducted the analysis with and moved Table 3 and the paragraphs commenting it to the Results section (Table 4 in the new version of the manuscript).**

*- Balanced-QPM development (Phase III) and Indicator structure design and performance measurement (Phase IV): Stay with the process (the method) and keep the results for the results section.*

**Thanks for this comment. In the new version of the manuscript, we have kept only the description of the methods used and moved all the results to the Results section.**

*RESULTS*

*Your results are interesting and relevant. Create a results section separate from the methods section.*

**Thanks for this suggestion. We have now a Results section.**

*DISCUSSION*

*The discussion needs to be further enriched and your results better compared with current knowledge (recent cases in literature and literature review).*

*From my point of view, the first two paragraphs of the discussion are still part of the results (implementation results).*

**You are right, we improved the discussion section following your recommendation.**

*- Future work: Ok, your opening is relevant.*

**Thanks.**

Some typos and errors:

*Introduction: “The Coordinating Committee, which is composed of a limited number of managers, aims at insuring” (ensuring?)*

 Right “ensuring”.

*Methods: “Due of this, it is challenging” (to)*

**Indeed, “to” is the correct term to use.**

*Conclusion: “which is has been reported in practice and in the literature as a very challenging problem”*

**Corrected, “which has been”.**

*In summary:*

*- The topic is interesting, and the study mobilizes a wealth of material to propose results relevant to the field.*

*- There are several study structuring issues that need to be addressed (structure of introduction, absence of results title, structure of discussion. A number of tips are given for this purpose.*

*- A number of methodological points need to be reinforced in order to fully convince the reader.*

*- The literature could be strengthened to develop the discussion on the contribution of this study. Proposals to this effect have also been made.*

*Good luck for the next steps.*

**Thanks for the relevant comments and suggestions that we believe significantly improved our manuscript.**
